# Transcriptomic characterization of *Trichoderma harzianum* T34 primed tomato plants: assessment of biocontrol agent induced host specific gene expression and plant growth promotion

**DOI:** 10.1186/s12870-023-04502-6

**Published:** 2023-11-08

**Authors:** Mohd Aamir, V. Shanmugam, Manish Kumar Dubey, Fohad Mabood Husain, Mohd Adil, Waquar Akhter Ansari, Ashutosh Rai, Pankaj Sah

**Affiliations:** 1 Division of Plant Pathology, ICAR-Indian Agricultural Research Institute, Pusa, New Delhi-110012, Delhi, India; 2 Department of Biotechnology, University Centre for Research & Development (UCRD), Chandigarh University, Punjab, 140413 India; 3 Department of Food Science and Nutrition, College of Food and Agriculture Sciences, King Saud University, Riyadh-11451, Saudi Arabia; 4 Plant, Food and Environmental Sciences, Dalhousie University, Truro, NS, B2N2R9 Canada; 5 Department of Botany, Centre for Advanced Study, Institute of Science, Banaras Hindu University, Varanasi, 221002 India; 6 Department of Basic and Social Sciences, College of Horticulture, Banda University of Agriculture and Technology, Uttar Pradesh, Banda, 210001 India; 7 Applied Sciences Department, College of Applied Sciences and Pharmacy, University of Technology and Applied Sciences-Muscat, Al Janubyyah Street, PO Box 74, Muscat, 133 Sultanate of Oman

**Keywords:** *Trichoderma*, Microarray, Transcriptional reprogramming, FDR correction, WCGNA analysis, DNA motif, Bottleneck genes, Hub genes

## Abstract

In this study, we investigated the intricate interplay between *Trichoderma* and the tomato genome, focusing on the transcriptional and metabolic changes triggered during the late colonization event. Microarray probe set (GSE76332) was utilized to analyze the gene expression profiles changes of the un-inoculated control (tomato) and *Trichoderma*-tomato interactions for identification of the differentially expressed significant genes. Based on principal component analysis and R-based correlation, we observed a positive correlation between the two cross-comaparable groups, corroborating the existence of transcriptional responses in the host triggered by *Trichoderma* priming. The statistically significant genes based on different *p*-value cut-off scores [(*p*_adj_-values or q-value); *p*_adj_-value < 0.05], [(*p*_cal_-values); *p*_cal_-value < 0.05; *p*_cal_ < 0.01; *p*_cal_ < 0.001)] were cross compared. Through cross-comparison, we identified 156 common genes that were consistently significant across all probability thresholds, and showing a strong positive corelation between *p*-value and q-value in the selected probe sets. We reported *TD2*, *CPT1*, pectin synthase, *EXT-3* (extensin-3), *Lox C*, and pyruvate kinase (PK), which exhibited upregulated expression, and *Glb1* and nitrate reductase (*nii*), which demonstrated downregulated expression during *Trichoderma*-tomato interaction. In addition, microbial priming with *Trichoderma* resulted into differential expression of transcription factors related to systemic defense and flowering including *MYB13*, *MYB78*, *ERF2*, *ERF3*, *ERF5*, *ERF-1B*, *NAC*, *MADS* box, *ZF3*, *ZAT10, A20/AN1*, polyol sugar transporter like zinc finger proteins, and a novel plant defensin protein. The potential bottleneck and hub genes involved in this dynamic response were also identified. The protein–protein interaction (PPI) network analysis based on 25 topmost DEG_S_ (*p*_cal_-value < 0.05) and the Weighted Correlation Gene Network Analysis (WGCNA) of the 1786 significant DEGs (*p*_cal-_value < 0.05) we reported the hits associated with carbohydrate metabolism, secondary metabolite biosynthesis, and the nitrogen metabolism. We conclude that the *Trichoderma-*induced microbial priming re-programmed the host genome for transcriptional response during the late colonization event and were characterized by metabolic shifting and biochemical changes specific to plant growth and development. The work also highlights the relevance of statistical parameters in understanding the gene regulatory dynamics and complex regulatory networks based on differential expression, co-expression, and protein interaction networks orchestrating the host responses to beneficial microbial interactions.

**Supplementary Information:**

The online version contains supplementary material available at 10.1186/s12870-023-04502-6.

## Introduction

The modern agricultural system is heavily dependent of synthetic pesticides, and chemical fertilizers to promote plant growth and development, improve productivity, and feed the rapidly growing world populations [1, 2]. Further, one of the biggest issues that modern agriculture faces is the challenges of meeting needs of the quality and quantity for the producer and customer without compromising environmental sustainability [3]. Nevertheless, extensive use of such fertilizers, chemicals, and other agricultural inputs has certainly affected the environment which directly or indirectly influences human health and the ecosystem. The need of the day is to minimize the usage of such cost-effective agricultural chemicals, develop alternative solutions for promoting ecological and environmental sustainability, foster a green economy, and increase the crop productivity through sustainable approaches [4]. With regard to ecological and environmental sustainability as well as fostering a green economy, the smart usage of beneficial plant–microbe interactions that have co-evolved during the course of evolution may be a useful strategy for feeding the world’s populations and providing plant-based food products [5]. In addition, the use of beneficial plant–microbe interactions has several advantages including increased seed germination, fertilizer use efficiency, and uptake of micro and macronutrients leading to improved plant growth, and development along with bonus effects like protection from various abiotic as well as biotic challenges [6–9]. *Trichoderma* fungi that are rhizosphere-competent are frequently utilized in commercial formulations as biofertilizers and biopesticides because of their numerous positive impacts on plant growth and disease resistance [10–12]. There are already a lot of products for biopesticides and biofertilizers on the market, most of which are based on useful symbionts of the genus *Trichoderma* [13, 14]. These fungi are well adapted to a plethora of ecological niches and well known for their ability to eliminate plant infections [15–17], as well as enhance plant growth and resilience to biotic and abiotic challenges [14, 18, 19]. According to reports, some *Trichoderma* strains have the ability to activate induced systemic resistance (ISR), a mechanism that is sparked by the root colonization of nonpathogenic rhizobacteria or fungi and controlled by a particular signal transduction cascade [14, 20]. Moreover, plants that have had certain *Trichoderma* isolates invade their roots become "sensitized" to pathogen assault and react more quickly and/or fiercely, through a process called priming [21, 22]. Several unanswered concerns about the biological basis of *Trichoderma's* effects have been studied using various 'omics-related methodologies [23]. *Trichoderma*-plant interactions have been studied using a variety of "omics" methods. Transcriptome investigations showed that root colonization differentially regulates the gene expression of the plant and its symbiont [24–26]. transcriptomic [27–30] and proteomic [31–33] approaches have been used to study the molecular mechanisms underlying plant responses to *Trichoderma* root colonization. Yuan et al. [34] reported the response of *T. longibranchiatum*-cucumber interaction and response by analyzing the host transcriptomic, proteomic, and phytohormonal content.

Recent research has shown that *Trichoderma* can boost plant development by either directly boosting the uptake of nitrogen and other nutrients by increasing root biomass or by solubilizing nutrients in soil [18, 35]. *Trichoderma* can control a wide range of plant pathogens by inducing induced systemic resistance (ISR) or localized resistance [19]. *Trichoderma* colonization of roots primes leaf tissues for increased activation of jasmonic acid (JA)-regulated defense responses, resulting in increased resistance to necrotrophic pathogens [36–39]. *Trichoderma* spp. produce enzymes and metabolites that can alter the plant's ethylene levels [40] and alter the structure of the roots [41–44], enhancing nutrient uptake. *Trichoderma* spp. promote plant growth through various indirect and direct mechanisms and induce systemic resistance against subsequent pathogen attack [45–50]. However, *Trichoderma*-inducedsystemic resistance (TISR) has been reported to involve multiple signaling routes, cross-communicating hormonal pathways, and networks that overall constitute a complex web of signaling cascades [39]. In addition, *Trichoderma* interaction with plants, or symbiosis, may also result in the expression of plant defense-related genes, which protect the plants from pathogens and thus aid in growth and development [25, 37, 51–56].

Transcriptional profiling or transcriptomic characterization using microarray technology is one of the most common approaches for identification and characterization of differentially expressed genes (DEGs). In fact, today, several commercial platforms are available and combined use of multiple platforms can overcome the inherent biases of each approach and this integrated approach may serve as a valuable complement to RT-PCR in discerning robust alterations in gene expression profiles [57]. Moreover, despite the advances in microarray technology, the array captures a large proportion of genes [58]. When it comes to expression data, there are numerous techniques to estimate the power of analysis [59–61]. The use of t-tests, ANOVAs, GO annotation, *p*-value cut-offs, Bonferroni corrections, array normalization, Fisher’s exact test, and fold change cut offs all result in a decrease in gene expression data, which may inadvertently limit or improve the power of study [58]. Hess and Iyer [62] reported that probe-level testing methods select many of the same genes as differentially expressed and therefore, suggested combined p is a promising alternative to existing methods of testing for differential gene expression.

In the last few years, several studies have been done on *Trichoderma*-host interaction or understanding the transcriptomic changes in different host tissues during early phases of interaction and colonization with fungus. Microarray analysis of *Trichoderma asperelloides* 203 interaction with *Arabidopsis* resulted in expression of transcription factors, stress-responsive genes, and transcriptional re-programming that modulated the host defense for initial host colonization [51]. Microarray analysis of *Arabidopsis thaliana* gene expression changes after 24 h of incubation in the presence of *T. harzianum* T34 using the Affymetrix GeneChip *Arabidopsis* ATH1 revealed the differential expression and downregulation of genes associated with SA and JA signaling while upregulation of several genes relevant to abiotic stress response was reported [30]. For example, colonization of cacao seedlings by four different endophytic *Trichoderma* isolates was studied to unravel the transcriptomic responses in both host and fungus during early colonization [27]. In one study, high-density oligonucleotide microarrays were used to study systemic modulation of gene expression by *T. hamatum* 382 in tomato just before the inoculation of pathogen *Xanthomonas euvesicatoria* [28]. The study reported the consistent modulation of gene expression by *T. hamatum* and the genes differentially expressed were associated with several categories including DNA, RNA, and protein metabolism, abiotic/biotic stress tolerance, plant defensins, particularly, PR5 proteins, extensin or extensin-like precursors [28]. Microarray analysis of early colonization of *T. asperelloides* 203 with *Arabidopsis thaliana* roots unraveled the differential expression of 137 genes related to stress response, transcription factors, and those involved in suppressing the host immunity for favoring the early colonization events [51]. Recently, a dynamic transcriptome study comprising of *T. virens*-maize interaction revealed the differential expression of genes in maize and were related to the biosynthesis of phytohormones and secondary metabolites, a wide array of cell wall degrading enzymes, genes related to shifting in metabolic activity and remodelling of cellular structures has been reported [63]. Similarly, RNA seq data for interaction of cucumber seedlings with *T. longibranchiatum* resulted into differential expression of genes related to secondary metabolism, defense/stress response, biosynthesis of phytohormones and signaling including ethylene (ET), jasmonic acid (JA), and salicylic acid (SA) [34]. These studies demonstrate that *Trichoderma* sp. induced transcriptional re-programming results into a complex molecular and physiological response resulting into altered hormonal signaling, enhanced nutrient ability, defense priming, and/or a potential cross-talk between growth promotion and defense pathways. However, a limited studies has been done regarding *Trichoderma*-host interaction post colonization events (3 weeks post colonization) as there is a huge gene-expression profile changes in transcriptome of both fungus and host tissues during early colonization events. The early colonization event of *Trichoderma*-tomato interaction is characterized by a wide gene transcript re-programming leading to enhanced defense response followed by transient suppression of host immune response to allow successful colonization [51]. However, once the fungus colonizes the host tissues changes in plant shoot transcriptome is not so much dynamic and the significant genes that are differentially expressed (statistically significant; *p*-value < 0.05) are also less than those that were involved during the early colonization event. These differentially expressed genes (DEGs), infact, are assumed to function as molecular trigger or biomarkers for different phenotypes [64]. Nevertheless, number of DEGs calculated from transcriptome data analysis could vary greatly when low quality samples were processed for data analysis or samples collected from a highly variable groups were incorporated into the study, regardless of different FDR-adjusted *p* value cut-offs [65].

The main objective of this study was to study the *T. harzianum* T34- induced transcriptional re-programming and understanding the molecular and biochemical changes as a rin host tissue during the late colonization event as a result of microbial priming. Our study and results aim to unravel the *Trichoderma harzianum* T34-induced transcriptional regulatory network involved in regulating plant growth and development and systemic defense. Transcriptional profiling of the un-inoculated tomato plants vs *Trichoderma* treated/primed plants uncovered the list of putative candidate DEGs that were sorted at different *p*-value (*p*_adj_-value < 0.05; *p*_cal_-value < 0.05, *p*_cal_-value < 0.01, and the *p*_cal_-value < 0.001) thresholds using two different statistical approaches like *p*-value based false positive rate and q-value based false discovery that were differentially expressed (based on FC values). The identified genes were further characterized for functional annotation and gene enrichment analysis. The topmost significant DEGs were analyzed for network analysis to predict the possible hub and bottleneck genes in a complex interactive protein–protein interaction network that regulated the biocontrol-induced metabolic alterations and crucial pathways involved in metabolic shifting, biochemical changes involved in favour of microbial priming induced plant growth and development during late colonization in the host. We further identified and characterized the gene family specific transcriptional factors that were significant and differentially expressed and therefore, regulating the *Trichoderma-induced*transcriptional re-programming and signaling cross-talk to fine tune the gene-regulatory network and gene-expression profile changes in the primed plants for enhanced stress tolerance, defense mechanisms, and growth promotion attributes.

This work presents a significant advancement in our comprehension of the intricate molecular mechanisms driving the influence of *Trichoderma*-induced modulation on tomato gene expression, particularly, during the later stages of the host colonization. Through the meticulous application of robust statistical analyses, a deeper insight has been gained into the intricate regulatory networks that govern the plant's responses to beneficial microbial interactions. By shedding light on metabolic pathways and gene specific biochemical alterations, this research not only enriches our understanding of plant–microbe interactions but also holds promise for shaping more sustainable and productive agricultural practices.

## Materials and methods

### Experimental Design for microarray analysis

For transcriptomic characterization of the differentially expressed genes across the tomato genome under the experimentally defined conditions (un-inoculated control vs *T. harzianum* T34 primed (treatment) through the microarray platform and the data generation, the submitter designed the experiments(GSE76332)[66]. In brief, tomato seeds *Solanum lycopersicum* L. “*Marmande*” variety and the fungal biocontrol *T. harzianum* CECT 2413 (Spanish Type Culture Collection, Valencia, Spain (that was referred as wilt type T34) was deployed [66]. For experimental design, the submitter primed tomato seeds with aqueous suspension 2 × 10^8^ spores mL^-1^ of *T. harzianum* T34 (1 mL of spore suspension/30 seeds) after surface sterilization with 2% sodium hypochlorite solution (20 min) followed by rinsing in a sterile distilled water. The seeds were then made to air dried in an open Petri plate overnight under a laminar flow hood following the protocol as suggested by Perez et al. [67]. Treated tomto seeds vs un-inoculated/un-primed seeds (control) were then made to sown in pots containing commercial loamy field soil that had been autoclaved at 121 °C for 1 h on two consecutive days. The data submitter defined the un-inoculated tomato seeds planted in sterilized pots as “Control” vs *T. harzianum* T34 primed seeds as “Treatment” GSE76332[66]. The pots were allowed to grow under the green house conditions at 22 ± 4 °C, and watered as needed. Three weeks later, tomato leaves were collected from two different cross-comparable groups (un-primed vs wild typeT34 primed tomato plants) and were allowed to keep immediately stored at -80 °C for RNA extraction and hybridization on Affymetrix microarrays.

### Data retrieval and correlation analysis

For data retrieval, we selected and studied the expression profile by using array probe sets submitted only for the uninoculated tomato plants (Control) vs *Trichoderma* T34 (WT) tomato interaction (treatment) available at NCBI with GEO profile ID GSE76332 [66]. To identify the significantly expressed genes with differential expression (genes are declared to be significantly expressed if an observed difference or change in read counts or expression levels between two experimental conditions is statistically significant (*p*_adj_-value < 0.05) with FC > 1 for upregulated; and the FC < 1 for down-regulated genes during interaction of *T. harzianum* (T34) with tomato plants. In this study, a total of two different cross-comparable probe sets each with three biological replicates including un-inoculated tomato control GSM1981375 (C1), GSM1981379 (C2), and GSM1981383 (C3) and the *Trichoderma*-tomato primed or treatment GSM1981376 (T1), GSM1981380(T2), and GSM1981384 (T3) from each array type were selected and analyzed, to understand the complex gene regulatory network regulating the physiological, biochemical changes and plant growth promotion effects in tomato during microbial priming with *T. harzianum* (T34). The microarray-based gene expression values for each and every probe set both from un-inoculated and tomato-*Trichoderma* interaction (treatment) was correlated using both the excel correlation plot method. Further, gene expression data were also correlated based on Pearson correlation coefficient values using tool R-4.2.2 (https://cran.r-project.org/bin/windows/base/) and the R studio software (https://support--rstudio-com.netlify.app/products/rstudio/download/).

### Microarray dataset analysis

A total of six samples of the array probe sets were selected and processed for GEO 2R analysis. GEO 2R analysis identified the genome-wide expression of the genes expressed in the tomato during *Trichoderma*-tomato interaction. For GEO2R analysis, NCBI GEO inbuilt Benjamini & Hochberg probability method [68, 69] was used for FDR correction and *p*-value adjustment (*p*_adjusted_-value) [68, 69]. To make sure that any biases introduced by the experiments have been eliminated, force normalization was applied [69, 70]. The significant level cut-off values were kept at their default settings (*p*-value ≤ 0.05). The DEGs were analyzed in the selected array probe sets within contrasting pair un-inoculated tomato Control (C1, C2, and C3) vs *Trichoderma*-tomato Treatment (T1, T2, and T3).

### Bioinformatics analysis

GEO 2R analysis was done to find the significant transcripts expressed genome-wide under the two contrasting groups based on their gene expression values. The expression data from both uninoculated control and *Trichoderma*-tomato treatment was analyzed for calculating the significant genes based on the Student's Ttest based experimentally calculated *p*-value, Fold change (FC), and significant genes that are differentially expressed (upregulated and downregulated). The Student's T-test was used for *p*-value calculation (to reject the null hypothesis) and obtaining the statistically significant genes. The parameters selected for the *p*-value calculations (*p*_calculated_-value) were array type: two tailed distribution and the paired data (control vs treatment). The IF logical function was used for sorting the significant genes keeping the parameter significant for *p*_cal_-value < 0.05 and not significant for data with *p*_cal_-value > 0.05. Further, significant genes were also retrieved at a lower cut-off score (*p*_cal_-value < 0.01) and even at a much lower stringent cut-off score (*p*_cal_-value < 0.001) to eliminate the false positive results [71–73]. The significant genes across the two cross-comparable experimental groups in the selected probe sets were also calculated and retrieved through FDR corrected and NCBI inbuilt Benjamini and Hochberg method derived *p*-value so called adjusted *p*-value (*p*_adjusted_-value) or q-value (FDR corrected *p*_adjusted-_value) and the results were further cross-compared with the experimentally calculated probability value (*p*_calculated_-value). To summarize the data (visually) and comparing the two experimental groups box-plot was used [74]. The volcano plot showing the significant DEGs (*p*_adj_-value < 0.05) both the upregulated (red dot) and downregulated genes (blue dot) was generated through eVITTA (https://tau.cmmt.ubc.ca/eVITTA/) [75]. The heat mapper tool (http://www.heatmapper.ca/) [76] was used to upload the expression data from individual expression values from each probe sets to display the differential expression of genes from two different contrasting pairs. The significant genes were sorted based on *p*_adj_-value < 0.05. The results obtained for finding the significant DEGs based on both FDR (*p*_adj_-value < 0.05) and without FDR (*p*_cal -_value < 0.05, *p*_cal-_value < 0.01 and *p*_cal-_value < 0.001) were sorted using the Venny 2.1 tool (https://bioinfogp.cnb.csic.es/tools/venny/) [77]. The custom vein diagrame was generated using the tool available at the (https://bioinformatics.psb.ugent.be/webtools/Venn/). Since genes that have more or less similar expression profile (co-expressed) are functionally associated or represent a part of similar complex or involved in a same pathway or regulatory mechanism or may influence each other or may be influenced by the same underlying mechanism(s). We analyzed the Weighted correlation network analysis (WGCNA) using the network module tool of the iDEP tool (http://ge-lab.org/idep/) [78] and the to construct a gene co-expression network derived for the significantly expressed genes (*p*_cal_-value < 0.05) across all the available probe sets and transcriptome samples. The soft threshold value was decided based on *R*^2^ value and was kept at 8 with minimum module size 16. The Clustvis tool https://biit.cs.ut.ee/clustvis/ [79] was used to show the differential expression of upregulated and downregulated genes with significant value (*p*_adj-_value < 0.05). The parameters for Clustvis analysis were analyzed using clustering distance for rows as Euclidean and clustering method for rows as average and tree ordering for rows analyzed with tightest cluster first. Likewise, the parameters kept for column were set as clustering distance for column as correlation and clustering method for column average and tree ordering as tightest cluster first with 1 cluster in column.

### Gene ontology and functional annotation

The accession identitities available with each specific probe sets were explored using NCBI- BLASTx tool in the non-redundant database (nr/nt). The topmost BLAST hits were further sorted based on their E-value, percentage identity, and the query coverages score values. The uncharacterized/hypothetical/ probable proteins were further analyzed for full length gene prediction. The identity of the protein was done based on sequential homology with the UNIPROT database (https://www.uniprot.org/) [80], using the BLAST algorithm [81] and applying an E < 10^−10^ level. The full length protein sequence was predicted and confirmed through the Sol Genomics Network server (https://solgenomics.net/) [82]. The full length gene prediction for the partial transcript/prpteins was identified and characterized using the Sol Genomics BLAST tool https://solgenomics.net/tools/blast/ [82] sequence with maximum identity (100%) and query cover (100%) values. The BLAST parameters kept for searching the protein across the category tomato genome current version and database tomato genome proteins ITAG 4.0. The protein sequence from the identified and characterized Soly IDs were further check using NCBI-BLASTp tool. The Affymetrix based probe identities for each gene were converted to gene identities using the DAVID tool (https://david.ncifcrf.gov/) [83, 84]. The identified protein sequence were further confirmed for their putative function using Phytozome Database https://phytozome-next.jgi.doe.gov/ [85]. The protein sequences were retrieved to find out the functional domain using PROSITE tool https://prosite.expasy.org/ [86]. The uncharacterized and hypothetical proteins without having any functional hits were identified based on their functional signature sequences using the tool INTERPROSCAN (https://www.ebi.ac.uk/interpro/) [87]. The highlighted sequences characterizing the functional domain were searched for possible mining of all the available isoforms of the proteins across the tomato genome using the tBlastn tool under database expressed sequence tags (ESTs) and were further sorted in our transcriptome data to identify the characterized the differential expression of putative isoforms of the similar protein across the two contrasting groups. The protein subcellular localization was predicted using Target P 2.0 tool https://services.healthtech.dtu.dk/service.php?TargetP-2.0 [88].

### Gene prediction

The identified and characterized proteins along with other hypothetical proteins were predicted using Fgenesch function of the softberry webserver http://www.softberry.com/. Gene prediction was done to find the structure of gene encoding TSS- transcription start (TATA-box position and score) and polyadenylation site (polyA), starting with start codon (CDS_f_), internal exon (CDSi), and CDS_l_—last coding segment, ending with stop codon). The BLASTx tool with non-redundant database nr/nt search was used to identify the available gene accessions associated with each specific probe. The proteins with less identities and query cover values were analyzed for full length gene prediction. For full length gene prediction the tBLASTn tool was used and the protein was searched across the whole genome sequence (WGS) platform for the investigated organism. The top hit accessions ids were selected for finding the position of full length ORF in the positive frame. The promoter region was searched 1500–2000 bases (depending on need) upstream to the transcriptional start site (CDS) and downstream to the stop codon or transcriptional termination site. The negative frame ORF was first converted to positive frame using the reverse complement tool (https://www.bioinformatics.org/sms/rev_comp.html). The nucleotide sequences were analyzed in the Fgenesch tool for finding the correct position of the available gene. The transcripts having multiple exons with exon–intron boundries were predicted using NCBI Gnomon tool (https://www.ncbi.nlm.nih.gov/genome/annotation_euk/gnomon/). To display the gene structure, position of intron, exons, and intron–exon boundaries gene structure display server GSDS 2.0 (http://gsds.gao-lab.org/) [89] was used for predicting the exact position and structure of the gene across the tomato genome. The gene structure of the predicted gene through Gnomon tool were further confirmed using the Augustus tool version 3.3.3 (https://bioinf.uni-greifswald.de/augustus/submission.php).

### DNA motif analysis

DNA motif analysis was performed to find the functional aspects of the consensus motifs associated with transcriptional factors. The common and specific transcriptional factor expressed differentially at *p*_cal_-value < 0.05, *p*_cal_-value < 0.01 and *p*_adj_-value < 0.01 predicted for finding the gene transcriptional regulatory sequence, transcriptional start site (TSS), and poly A tail. All the members expressed across the tomato genome under the *Trichoderma* treatment condition were predicted and processed for finding the consensus DNA motif using MEME suite tool (https://meme-suite.org/meme/) [90]. Further, the functional aspects of the motifs were determined using TOMTOM tool (https://meme-suite.org/meme/tools/tomtom) [91].

### Protein–protein interaction and network analysis

Protein–protein interactive association network was explored using STRING version 11.5 https://string-db.org/ [92, 93] to unravel the probable protein partners associated with our target genes and proteins. The K clustering algorithm was deployed to specify the functional annotation in separate number of clusters, and therefore, distinguishing proteins with high interacting global score values constituting one cluster from a complex network of multiple interacting proteins along with the dashed-lines denoting inter-cluster edges. The nodes were distributed based on their GO ontological terms to specify the shared functions contributed by each proteins from a group of interactive associative network. PPI enrichment value denoted the relevance of interaction network based on their individual strength, FDR correction, and network count for each specific protein from each group. The entire network was then exported to Cytoscape for comprehensive assessment of the molecular signaling pathways and prediction of associated gene-regulatory network through network analyzer tool for directed graph. The network analyzer analyzed the complex interaction based upon number of nodes (genes), edges, clustering co-efficient, degree of interaction, betweeneess centrality, network density, closeness centrality, and average shortest path to determine the major hub and bottleneck genes in the gene-regulatory network. The directed graph showing the top 10 hub and bottleneck genes in the gene-regulatory network were determined based on Cytohubba plugin of the Cytoscape https://cytoscape.org/ [94]. The Cytohubba plugin characterized the interactive associative network based on degree of interaction, bottleneck, and closeness parameters identifying putative hub bottleneck node, non-hub bottleneck node, hub non-bottleneck node and non-hub bottleneck nodes associated with protein interactive networks. The functional annotation of the characterized DEGs with significant functional enrichment values along with their GO identities were characterized for their functional aspects using AmiGO2 tool (http://amigo.geneontology.org/amigo) [95] and further validated using the tool QuickGO (https://www.ebi.ac.uk/QuickGO/) [96]. The identified and characterized DEGs were further analyzed using ShinyGO (http://ge-lab.org/go/) [97] for GO enrichment analysis. The functional annotation and gene-specific pathway were retrieved through ShinyGO based KEGG tool [98, 99].

## Results

### Tomato genes expression during *Trichoderma* interaction and colonization

#### Data retreival, correlation, and GEO data analysis

**Fig. 1:**
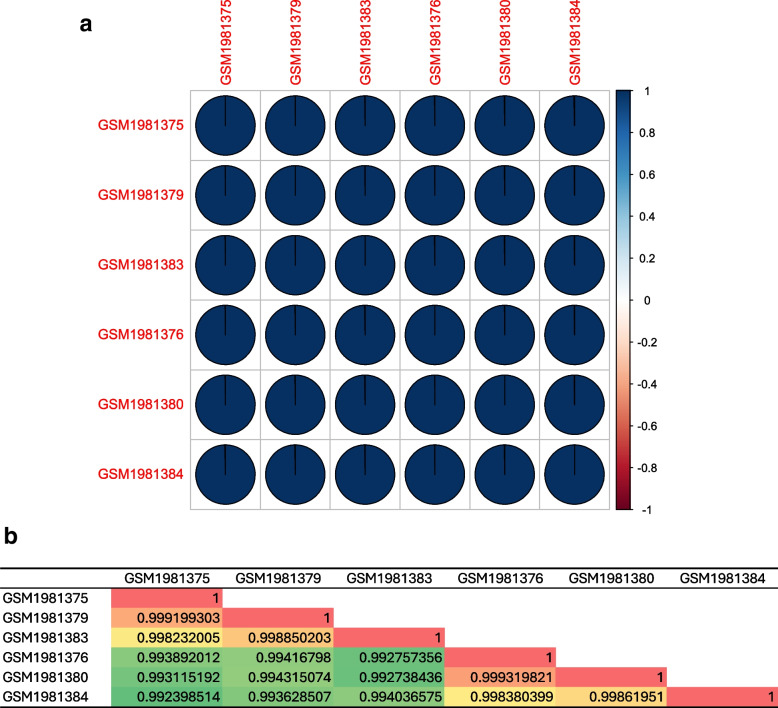
**a** The R-based correlation pie chart showing the positive correlation between the two cross-comparable experimental datasets including the un-inoculated control (GSM1981375, GSM1981379, and GSM1981383) and the *Trichoderma T34*-tomato interaction (GSM1981376, GSM1981380, and GSM1981384). **b** The Excel correlation plot showing the positive correlation between the un-inoculated control (C1, C2, and C3) and *Trichoderma T34*-tomato treatment (T1, T2, and T3). For correlation plot significant genes at lower p_cal_-value (p_cal_<0.01) were selected for correlating the gene expression array datasets

**Fig. 2:**
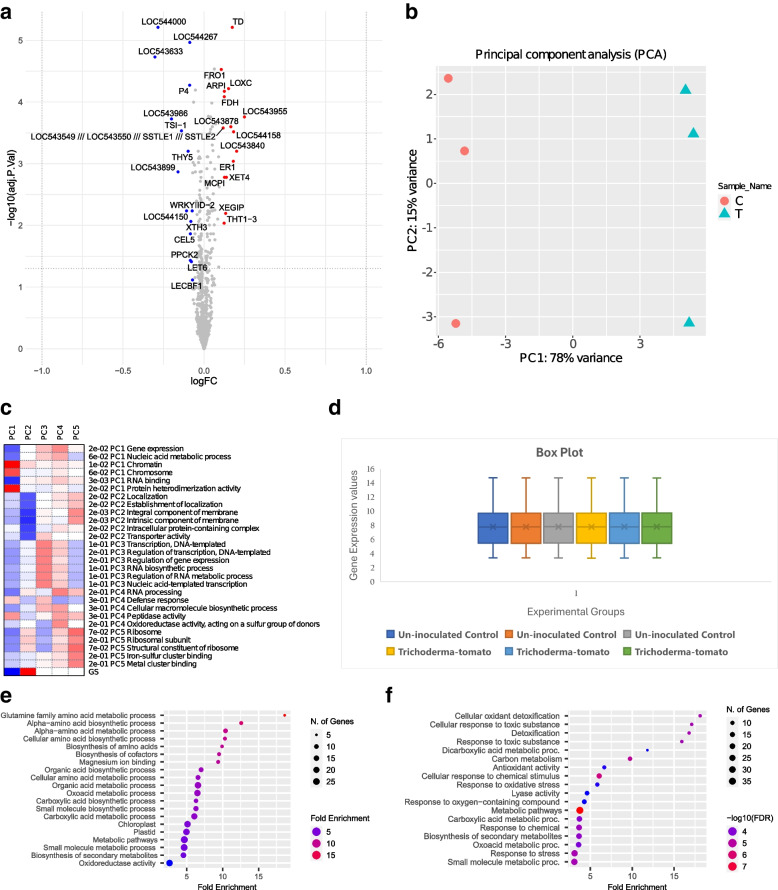
**a** The Volcano plot showing the significant and differentially expressed genes (*P*_cal_ < 0.05). The Y axis denotes the mean expression value of log 10 (*p*-value) whereas the X axis displays the log2 Fold change value. The highlighted genes are significantly expressed at a default adjusted *p*-value cut-off of 0.05 (red; denotes upregulated, blue; denotes downregulated). Log FC indicates the log of the change in gene expression in *T. harzianum* T34 primed treatments compared to the un-inoculated control. Log counts are the log of the read counts mapping to each gene. Volcano plots show the LogFC vs. the – log10 FDR (False discovery rate), points with more fold change and higher –log10 FDR are more reliable. **b** PCA analysis plot for tomato un-inoculated control (C1, C2, and C3) and the treatment *Trichoderma*-tomato interaction (treatment) (T1, T2, and T3). **c** PCA heat map showing the pathway analysis of PCA rotation for the significant DEGs (*p*_cal_-value < 0.05). **d** The Box-plot showing the distribution showing the spread and centers of a data set. Measures of spread include the interquartile range and the mean of the data set. Measures of center include the mean or average and median (the middle of a data set). **e** Enriched GO annotation for the down-regulated DEGs (*p*_cal_-value < 0.05) showing the gene-specific pathways and number of significant genes (enrichment-specific to a particular pathway based on data from Ensemble plant and other databases. **f** Enriched GO annotation for the Up-regulated genes

The individual expression value associated with each array datasets were correlated using R and the R bases correlation graph showing the positive correlation between the un-inoculated control (C1, C2, and C3) and the *Trichoderma*-tomato (T1, T2, and T3) (Fig. 1a) and the same has been verified using excel based correlation graph (Fig. 1b). Genome wide analysis of the micro-array based transcriptome data unravelled the list of total genes expressed in two different array probe sets under the defined conditions. The GEO2R analysis performed with uninoculated control (C1, C2, and C3) and the *Trichoderma*-tomato treatments (T1, T2, and T3) identified the total 10209 genes expressed during *T. harzianum* priming induced transcriptional re-programming in tomato during *T. harzianum* T34 colonization. The volcano plot showed showed statistical significance (-log10 *P* value) versus magnitude of change Log 2 fold change (Log 2FC) for all the differentially expressed significant genes (Fig. 2a). Based on distribution of the hits as found on volcano plot some of the significant (*p*_cal_-value < 0.05) with downregulated expression for statistically significant genes (*p*_cal_-value < 0.05) hits found in blue dots (down-regulated) were phosphoenol pyruvate carboxykinase (PPCK2), glutamine synthase (GTS1), cellulase (CEL5), xyloglucan endo transglucosylase/hydrolase (XTH3), WRKY transcription factor II-d, and P4 pathogenesis related protein, homeobox protein knotted1 like (LET6). In contrast, the hits assigned with red dots (upregulated expression profile) were phospholipase D (PLDA2), lipooxygenase C (Lox C), ferric chelate reductase (FR6-1), xyloglucan specific fungal endoglucanase inhibitor protein (Xe GIP), formate dehydrogenase (FDH), threonine dehydratase (TD), LRR receptor like serine threonine kinase, N-hydroxy cinnamoyl transferase (THT1-3), metallo carboxypeptidase inhibitor (MCP1), allene oxide synthase. The principal component analysis graph showing the positive correlation between the two experimentally different groups including the uninoculated control samples (C1, C2, and C3) and the *Trichoderma*-tomato treated samples (T1, T2, and T3) (Fig. 2b). The PCA plot showed 78 percnt variance in PC1 group and 15 percent variance in PC2 group. The PCA table based on individual PCA groups, multidimensional scaling, t-distributed stochastic neighbor embedding (t-SNE) has been shown in Table S1. The PCA plot indicated that a cumulative variance (PC1 + PC2) of 93 percent in the transcriptomic data is due to the two selected arry probe sets and a positive correlation between the two different experimental conditions including un-inoculated control versus *Trichoderma*-tomato treatments. The PCA heatmap showing the pathway analysis of the PCA rotation (Fig. 2c). The Box plot digrame showed median centered distribution of expression values indicating the expression data is normalized and cross-comparable in both array probe sets (Fig. 2d). The multidimensional scaling plot for the selected 1786 DEGs (*p*_cal_ < 0.05) has been shown in (Fig. S1). The two-dimensional t-distributed stochastic neighbor embedding (t-SNE) plot for uninoculated control vs *Trichoderm*a-tomato treatment has been shown in (Fig. S2). The pathway analysis of the PCA rotation for the GO terms biological processes (Fig. S3), molecular function (Fig. S4) and KEGG pathways [98, 99] (Fig. S5) has been shown. The gene set enrichment analysis showing the functional annotation of the significantly expressed and upregulated DEGs (Fig. 2e) and downregulated DEGs (Fig. 2f).

### DEGs bioregulation analysis

**Fig. 3:**
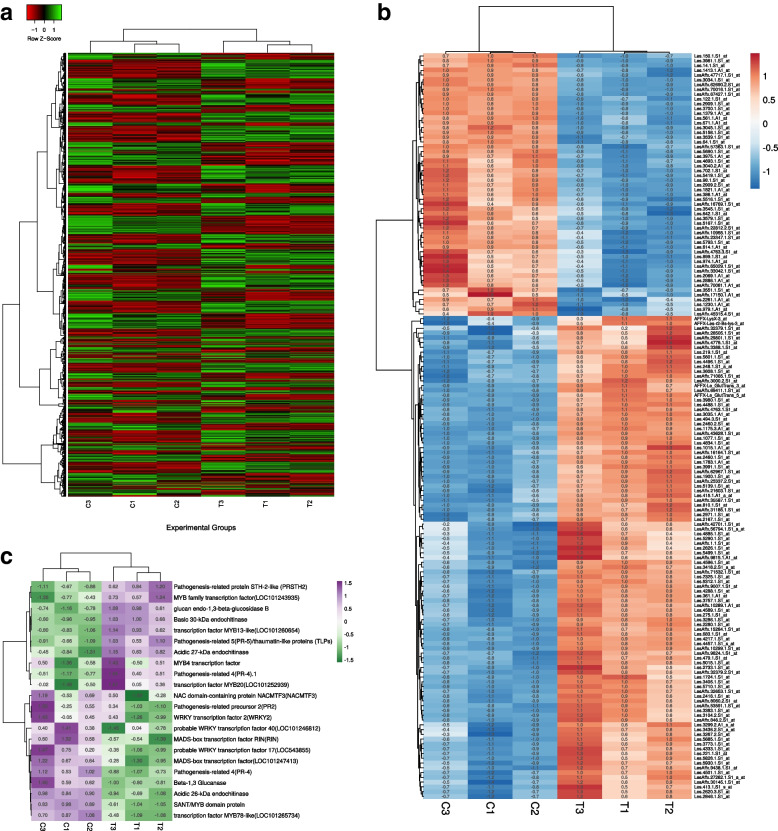
**a** Heat map based on clustering of the multivariate data of the two selected cross -comparable groups and datasets including tomato un-inoculated control (GSM1981375, GSM1981379, and GSM1981383) and *T. harzianum* T34 primed (treatment) tomato plants (GSM1981376, GSM1981380, and GSM1981384). **a** Heatmap showing the clustering of 1786 differentially expressed and significant genes (*P*_cal_ < 0.05) and the heat map was derived through the heatmapper tool. The significant genes that are differentially expressed have been sorted based on fold change with upregulated genes (FC > 1) and down-regulated genes (FC < 1). **b** Heat map showing the clustering of the 156 DEGs based on their individual expression values and were sorted based on their significant *p*-value cut-off scores. These 156 DEGs were reported to be present in all the probability groups (*p*_cal-_value < 0.05; *p*_cal_-value < 0.01, and the FDR corrected *p*_adj_-value < 0.05). The rows and columns have been clustered using the average method. The row distance was decided using the correlation approach and the columns have been clustered using the Eucilidean approach. **c** Figure showing the heat map for the differential expression of the PR proteins and various transcription factors across the two cross-comparable experimental groups

**Fig. 4:**
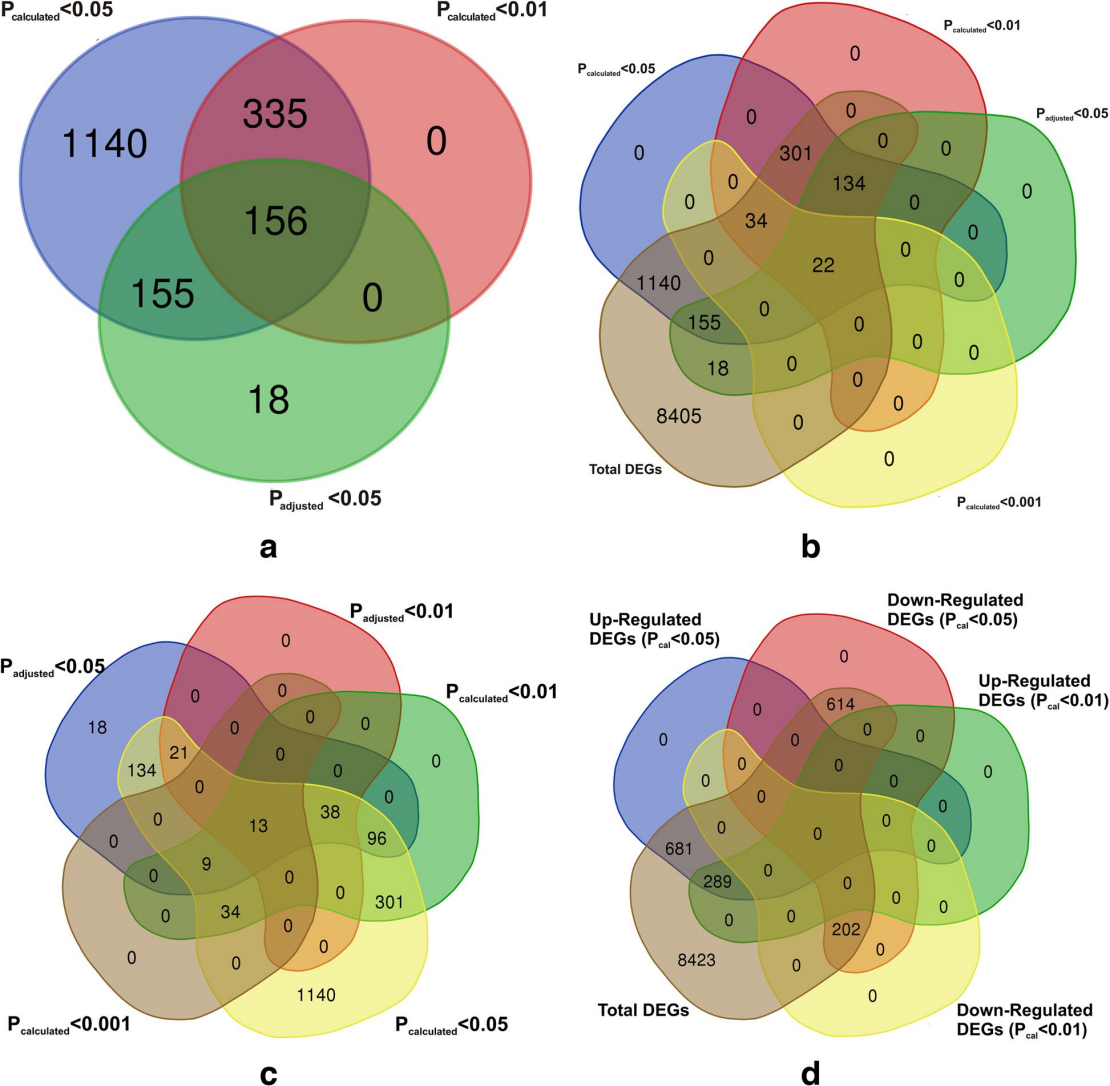
The vein digrames showing sorting of the significant genes based on experimentally calculated (Student's T Test and the FDR adjusted probability score values. **a** The vein digramme showing the proportion of common 156 genes sorted based on *p*_adj_-value < 0.05, *p*_cal_-value < 0.05, and *p*_cal_-value < 0.01). **b** The Vein digramme showing the proportion of shared 13 significant genes differentially expressed based on cross comparision of FDR corrected and FDR underestimated *p*-values and at much more stringent-cut-off scores of the experimentally derived *p*-values. **c** The vein digramme showing the proportion of significant 22 genes out of the total genome-wide expressed genes and were sorted based on *P*_cal_ < 0.05, *P*_cal_ < 0.01, *P*_adj_ < 0.05, and too much stringent and lower cut-off score values of experimentally derived raw data based *P*_cal_ < 0.001values. **d** The Vein digrammes showing the proportion of the significant DEGs (both upregulated and the downregulated) across the two cross-comparable groups (un-inoculated control vs *T. harzianum* T34 primed tomato plants) and were compared to the total genome-wide expressed genes acoss the tomato genome under the defined experimental conditions. The data was filtered at *P*_cal_ < 0.05 and *P*_cal_ < 0.01 and the FDR corrected *P*_adj_ < 0.05

Generally, gene expression patterns are able to provide essential cues for gene function. In our result, out of total 10,209 genes expressed across the tomato genome under two cross-comparable defined groups in tomato array probe sets, we found 329 significant genes (*P*_adj_ < 0.05) with differential expression expression in two contrasting cross-comparision groups (uninoculated tomato and tomato treatment with *T. harzianum* T34. However, genes retrieved based on Student Ttest based probability value calculations (p_calculated_) using experimentally derived individual expression values for data sets reported a total of 1786 significant DEGs (*p*_cal_-value < 0.05) and a total of 491 DEGs at a lower and stringent cut-off scores (*p*_cal_-value < 0.01) avoiding FDR corrections. The heat map showing the differential expression of 1786 genes across the two different treatments (CI, C2, and C3), and (T1, T2, and T3) has been shown in (Fig. 3a). The heat map showing the genome wide expression of 156 common DEGs in all the probability groups have been shown (Fig. 3b). The heat map showing the differential expression of all the PR proteins, transcription factors at all the probability groups (*p*_cal_-value < 0.05; *p*_cal_-value < 0.01; *p*_adj_-value < 0.05) (Fig. 3c). Interestingly, sorting of the significant DEGs retrieved through the FDR corrected *p*_adj_-value (*p*_adj_-value < 0.05) and genes from two different calculated *p*-values with underestimating the FDR correction (*p*_cal_ -value < 0.05 and *p*_cal-_value < 0.01) we obtained 156 DEGs common in all the three groups (*p*_adj-_value < 0.05, *p*_cal_-value < 0.05 and *p*_cal_-value < 0.01) with 335 common elements in between *p*_cal-_value < 0.05 and *p*_cal_-value < 0.01 and also reported the same 156 common elements present between *p*_adj_-value < 0.05 and *p*_cal_-value < 0.01 (Fig. 4a). Interstingly, sorting of the genes based on FDR corrected *P*_adjusted_ values at a cut-off score *P*_adj_ < 0.05 and even at a stringent cut-off value of *P*_adj_ < 0.01 and their further comparision with experimentally derived p_calculated_ cut-off score values (*P*_cal_ < 0.05, *P*_cal_ < 0.01, and even at a much lower stringent cut-off score values *P*_cal_ < 0.001 revelaed 13 common DEGs (Fig. 4b). Nevertheless, sorting of significant genes at a much more lower stringent cut-off score value (*p*_cal_-value < 0.001) and its comparision with FDR calculated *p*_adj_-value < 0.05 and two other experimentally derived raw *p*-values (without FDR correction) or calculated probability values (*p*_cal_-value < 0.05 and *p*_cal_-value < 0.01) reported 22 significant genes common in all the probability groups (Fig. 4c). The differential expression of significantly up-regulated genes at all the probability groups (*p*_adj_-value < 0.05, *p*_cal_-value < 0.05. and *p*_cal_-value < 0.01) has been shown in Table 1. The differential expression of significantly down-regulated genes at all the probability groups (*p*_adj_-value < 0.05, *p*_cal_-value < 0.05. and *p*_cal_-value < 0.01) has been shown in Table 2. However, we found only 155 common elements were reported to be present in *p*_adj_-value < 0.05 and *p*_cal-_value < 0.05.

**Table 1:** List of top most significant genes encountered in all the probability groups (*p*_cal_ < 0.05, *p*_cal_ < 0.01, and *p*_adj_ < 0.05 and were differentially expressed and upregulated (*p*_cal_-value < 0.05; FC>1) in all the biological replicates (T1, T2, and T3) of *Trichoderma*-tomato interaction samples compared to all the un-inoculated (C1, C2, and C3) control samples. The results were further validated using the Sol Genomics BLAST tool for the identification and characterization of the specific isoform of the protein

Probe ID	Putative Identity	Sol Genomics ID	Fold Change	*p*-value	Functional Category	Putative Function
Les.1077	ATP dependent Clp protease	Solyc02g078160.3.1	1.297	1.45E-04	Stress Response	Protein Degradation through ATP dependent ATPases
Les.415	Neryl diphosphate synthase 1(CPT1)	Solyc08g005680.4.1	1.224	5.63E-04	Secondary Metabolism	Terpene Biosynthesis
Les.4488	Threonine dehydratase biosynthetic, chloroplastic(TD2)	Solyc09g008670.3.1	1.137	3.93E-03	Protein Metabolism	Isoleucine Biosynthesis
Les.4885	BAHD acyltransferase DCR(LOC101256185)	Solyc05g052670.1.1	1.131	2.90E-03	Secondary Metabolism	Biosyntheiss of Hydrocinnamic esters
Les.2460	organ-specific protein S2(LOC101245207)	Solyc10g009150.3.1	1.129	1.98E-03	Defense Response	Serine proteases degrading peptide bonds from pathogen proteins
LesAffx.32379	polygalacturonate 4-alpha-galacturonosyltransferase (pectin synthase)	Solyc04g079860.1.1	1.128	2.38E-03	Carbohydrate metabolism	Biosyntheis of Pectic compounds
Les.3980	lipoxygenase (loxC)	Solyc01g006540.4.1	1.128	3.52E-03	Plant Growth, Development, and Defense	Generation of Fatty Acid-Derived Flavor Compounds
LesAffx.9007	Uncharacterized	Unknown	1.126	5.24E-03	Not Known	Not Known
Les.4288	pyruvate kinase 1, cytosolic (LOC101248036)	Solyc09g008840.4.1	1.124	3.78E-03	Carbohydrate metabolism	Glycolytic pathway
LesAffx.71065	Protein Early Flowering 2-like (ELF2-like)	Solyc04g064870.3.1	1.112	1.32E-03	Flower development and Stress reponse	AP2/ERF type transcription factor involved in flower development, hormonal signaling and stress response
LesAffx.3388	Plant cell wall protein SlTFR88	Solyc10g074730.3.1	1.112	2.69E-03	Stress response and Plant Defense	hydroxyproline-rich glycoproteins (HRGPs) involved in cell expansion, differentiation,cell structure, and plant microbe interactions
Les.1783	kirola(LOC101262669)/Bet v I/Major latex protein domain-containing protein	Solyc10g048030.2.1	1.109	1.47E-03	Plant Defense and Stress Response	Plant Defense and Stress Response
Les.4457	Sesquiterpene synthase 1 (SSTLE1)	Solyc06g059930.4.1	1.104	7.42E-04	Secondary Metabolism	Biosynthesis of Terpenes involved in Plant Defense against pathogens, and herbivores
Les.275	formate dehydrogenase(fdh)	Solyc02g086880.4.1	1.103	2.81E-03	Energy Metabolism and Cellular Homeostasis	Formate metabolism and CO2 fixation
Les.361	Beta-phellandrene synthase(chloroplastic)	Solyc08g005677.1.1	1.098	3.57E-03	Plant Defense, Stress Response	Biosynthesis of beta-phellandrene(terpene synthase)
LesAffx.30145	Plant Cadmium Resistance 2 (PCR2)	Solyc01g005470.3.1	1.094	3.82E-03	Plant Defense, Stress Response	Cd detoxification and sequesteration, defense against oxidative stress
LesAffx.32379	Probable galacturonosyltransferase-like 1(LOC101247911)	Solyc04g079860.1.1	1.092	3.53E-03	Carbohydrate metabolism	Biosyntheis of Pectic compounds
Les.5139	Epidermis-specific secreted glycoprotein EP1(LOC101268888)	Solyc07g062490.1.1	1.087	5.61E-03	Cell adhesion, signaling, and/or immune responses	member of the Beta-microseminoprotein family involve in cell structure, adhesion and immune response
LesAffx.28505	Perakine reductase(LOC101256520)	Solyc09g082720.3.1	1.086	6.17E-03	Secondary Metabolism	Biosynthesis of the alkaloid perakine
Les.1900	Kirola (LOC101264616)	Solyc04g007760.3.1	1.084	7.58E-04	Plant Defense and Stress Response	Plant Defense and Stress Response
Les.3035	Aspartic protease inhibitor 1(LOC101262903)	Solyc03g098780.2.1	1.084	5.41E-03	Protein Metabolism	Protein degradation
LesAffx.53591	Transcription factor MYB13-like (LOC101260654)	Solyc06g083900.3.1	1.081	1.82E-03	Secondary Metabolism	Anthocyanin Biosynthesis
LesAffx.4763	MADS-box transcription factor(LOC101247413)	Solyc12g087830.2.1	1.08	8.26E-03	Plant growth and Development	Flower formation, fruit ripening, and seed germination
Les.3286	Inducible plastid-lipid associated protein	Solyc07g064600.3.1	1.078	4.23E-03	Lipid Metabolism	Synthesis and accumulation of lipids in response to stress response
Les.2460	Organ-specific protein S2(LOC101245207)	Solyc10g009150.3.1	1.071	6.26E-04	Protein Metabolism	Seine proteases function as a scaffold protein for cellular structure re-organization
Les.5685	High-affinity nitrate transporter	Solyc02g083120.3.1	1.071	3.85E-03	Nitrogen metabolism	Nitrate uptake and transport
Les.1015	Dehydrodolichyl diphosphate synthase 2(CPT7)	Solyc06g076920.3.1	1.07	7.83E-03	Protein Metabolism	Biosynthesis of Dolichol, a polyisoprenoid alcohal for protein glycosylation
LesAffx.35587	Vacuolar processing enzyme VPE3 precursor	Solyc08g065610.2	1.069	6.82E-03	Programmed cell Death and Apotosis	cysteine proteases involved in processing of stored food during seed germination
Les.3267	Bifunctional UDP-glucose 4-epimerase and UDP-xylose 4-epimerase 1(LOC101266745)	Solyc08g080570.4.1	1.064	9.90E-03	Carbohydrate metabolism	Biosynthesis of various cell wall polysaccharides, including xyloglucan and xylan
LesAffx.1.1.S1_at	Arginase 2	Solyc01g091170.3.1	1.064	3.39E-03	Protein and Nitrogen metabolism	Degradation of protein and other nitrogen compounds
Les.2971	Wound-induced proteinase inhibitor 1(LOC101246961)	Solyc09g084440.2.1	1.063	4.64E-03	Plant Defense against herbivores and pathogen	Kunitz-type inhibitors function as proteinase inhibitor
Les.1175	Uncharacterized protein	Solyc03g025670.3.1	1.062	6.48E-03	Unknown	Unknown
LesAffx.4779	Peroxidase 12(LOC101253377)	Solyc04g071890.3.1	1.058	9.54E-03	Plant Defense	Degradation of toxic substances and host defense
LesAffx.71532	NBS-LRR type receptor like protein33(RLP33)	Solyc10g052880.1.1	1.053	9.29E-03	Plant Defense	Plant defense. particularly, against fungal pathogens
Les.3436	Metallocarboxypeptidase inhibitor	Solyc06g061230.3.1	1.051	5.74E-03	Protein Metabolism	Breakdown of proteins and peptides
LesAffx.9438	WD repeat-containing protein 43(LOC101255780)	Solyc03g116380.4.1	1.049	2.20E-03	DNA and RNA metabolism	RNA metabolism, protein transport, and chromatin remodeling
Les.3757	Glutaminase domain-containing protein(LOC544312)	Solyc03g006490.3.1	1.049	6.91E-04	Amino acid metabolism	Plant growth and development, nitrogen assimilation, and stress response
Les.5499	Probable nucleoredoxin 2	Solyc04g081900.4.1	1.049	1.42E-03	Plant Growth and Development	Photosynthesis, plant growth, and responses to environmental stress
LesAffx.29801	Fe_2_OG dioxygenase domain-containing protein	Solyc03g080190.3.1	1.049	8.22E-03	DNA and RNA metabolism	DNA repair, RNA metabolism, binding of iron and oxygen molecule, and synthesis of various metabolites
Les.1724	Glutathione S-transferase L3 isoform	Solyc10g084400.2.1	1.047	9.27E-03	Plant defense and Stress Response	Detoxification and metabolism of various toxins, and environmental factors leading to oxidative stress
Les.5710	BTB/POZ domain-containing protein	Solyc11g012840.2.1	1.046	6.05E-03	Stress Response	Fruit development and response to stress
LesAffx.9824	DMR6-LIKE OXYGENASE 2-like (LOC101260084)	Solyc01g105660.4.1	1.045	4.45E-03	Cell Death and Stress Response	Regulation of the jasmonic acid (JA) signaling pathway
Les.3608	Peroxidase(CEVI-1)	Solyc01g006300.3.1	1.043	1.01E-03	Plant Stress Response	Cellular redox balance and protection against oxidative stress
Les.413	Glutathione S-transferase/peroxidase(BI-GST/GPX)	Solyc07g056480.3.1	1.042	6.40E-03	Plant Defense	Defense against Geminiviruses
Les.5601	Protein SRG1	Solyc02g071380.2.1	1.041	7.25E-03	Plant Defense	Plant defense against both Biotic and Abiotic stress
LesAffx.42701	Nicotinamidase 1	Solyc01g106000.4.1	1.041	7.97E-03	Plant Defense	Plant Defense
Les.4496	Pathogenesis-related protein STH-2-like(LOC101246381)	Solyc09g090980.3.1	1.039	8.79E-03	Cellular Homeostasis	Protein folding and molecular chaperon
LesAffx.9815	DNAJ-like protein(DNAJ)	Solyc04g081530.1.1	1.039	8.96E-04	Plant growth and Development	Seed Dormancy and Germination
LesAffx.15284	Auxin repressed/dormancy associated protein(LOC101258429)/DRM1/ARP	Solyc02g077880.3.1	1.037	5.76E-03	Plant Growth, Development and Defense	Defense response to biotic stress, including salicylic acid (SA) and methyl jasmonate (MeJA) signaling
Les.4834	Serine decarboxylase(LOC101262912)	Solyc04g071140.3.1	1.026	4.94E-04	Amino acid metabolism	Phospholipid and amino acid metabolism in A. thaliana
LesAffx.10299	Polyol transporter 5-like(PMT5)	Solyc01g109460.3.1	1.024	5.42E-05	Plant growth and Development	plasma membrane hexose sugar transporters in A. thaliana

**Table 2:** List of top most significant genes encountered in all the probability groups (*p*_cal_ < 0.05, *p*_cal_ < 0.01, and *p*_adj_ < 0.05) and were differentially expressed and down-regulated (*p*_cal_-value < 0.05; FC <1) in all the biological replicates (T1, T2, and T3) of Trichoderma-tomato interaction samples compared to all the un-inoculated (C1, C2, and C3) control samples. The results were further validated using the Sol Genomics BLAST tool for the identification and characterization of the specific isoform of the protein

**Table 2 Probe ID**	**Putative Identity**	**Sol Genomics ID**	**Fold Change**	***p*-value**	**Functional Category**	**Putative function**
**Les.1413**	Malate dehydrogenase, chloroplastic(LOC101258932)	Solyc03g115990.3.1	0.982	1.50E-03	Carbohydrate metabolism	Citric acid cycle and energy production in plants
**Les.3975**	Sulfite reductase(LOC101055609) sir	Solyc11g065620.2.1	0.981	6.40E-03	Sulphur metabolism	Sulphur assimilation
**LesAffx.47717**	Uncharacterized protein	Solyc01g081480.3.1	0.98	8.17E-03	Not Known	Not Known
**Les.5690**	Soluble inorganic pyrophosphatase 6, chloroplastic(LOC101264469)	Solyc10g047950.2.1	0.978	3.60E-04	Plant Growth and Development	ATP synthesis, Photophosphorylation
**Les.5189**	Asparagine synthetase [glutamine-hydrolyzing] 2(LOC101259236)	Solyc04g055200.3.1	0.978	9.04E-03	Nitrogen Metabolism	Biosynthesis of asparagine from the glutamine
**Les.5161**	Interactor of constitutive active ROPs 2, chloroplastic(LOC101255915)	Solyc09g007360.3.1	0.978	4.50E-03	Stress Response	Plant-specific GTPases playing a critical role in cytoskeleton organization, hormone signaling, preventing photooxidative damage to chloroplast
**Les.14**	Subtilisin-like protease SBT2(SBT2)	Solyc03g006970.1.1	0.977	5.66E-03	Plant Growth, Development, and Defense	Processing and activation of several precursor proteins required for plant development
**LesAffx.13831**	Plastid division protein PDV2(LOC101261370)	Solyc01g109260.3.1	0.977	3.08E-03	Plant Growth and Development	Division of plastids
**Les.98.1**	MAR-binding filament-like protein 1(MFP1)	Solyc03g120230.3.1	0.976	6.96E-03	Plant Growth and Development	Nuclear architecture and genome organization
**Les.1230**	L-ascorbate peroxidase 3(LOC101264282)	Solyc02g083630.3.1	0.968	5.29E-04	Stress Response	ROS scavenging and Cellular Homeostasis
**Les.3579**	Type 2 metallothionein MT3(MT3)	Solyc04g058100.3.1	0.968	4.99E-03	Cellular Homeostasis	Metal ion homeostasis, detoxification, and regulation of gene expression
**Les.64**	Gibberellin 20-oxidase-1(GA20ox1)	Solyc03g006880.3.1	0.966	1.58E-03	Plant Growth and Development	Biosynthesis of Gibbrellins, seed germination, stem elongation, and flower and fruit development
**Les.386**	Probable protein phosphatase 2C 52(LOC101259375)	Solyc10g008490.3.1	0.966	2.93E-03	Plant Growth, Development and Stress response	Regulation of cell cycle, hormonal signaling, and stress response
**Les.561**	Protein PIN-LIKES 3-like(LOC101247117)	Solyc12g095750.2.1	0.962	7.56E-03	Cell Metabolism and Hormonal signaling	Auxin transport and signaling
**Les.702**	S-adenosylmethionine decarboxylase 2(LOC101260400)	Solyc02g089610.2.1	0.961	4.37E-04	Plant Growth and Development	Chromatin remodeling and RNA processing, and biosynthesis of polyamines and spermidines
**Les.122**	Acidic 26 kDa endochitinase (Chitinase CHI3)	Solyc02g082920.4.1	0.955	6.00E-03	Plant Defense	Plant defense against fungal pathogens
**Les.1379**	Hop-interacting protein THI116(LOC101055532)	Solyc03g117250.4.1	0.954	3.61E-03	Stress Response	Co-chaperon regulating the activity of molecular chaperones Hsp70 and Hsp90
**Les.642**	Probable alkaline/neutral invertase B(LOC101253328)	Solyc01g111100.5.1	0.942	5.10E-03	Carbohydrate metabolism	Seed germination and fruit ripening
**Les.3981**	UDP-glycosyltransferase 76E1(UGT76E1)	Solyc10g085230.2.1	0.942	3.12E-03	Plant cell Metabolism and Defense	Glycosylation of various natural compounds, including flavonoids, alkaloids, and terpenoids
**LesAffx.67427**	Protein RESPONSE TO LOW SULFUR 3-like(LOC101243684)	Solyc03g096770.1.1	0.941	4.86E-04	Plant Growth and Development	Sulphur metabolism
**Les.3045**	Adenylyl-sulfate reductase(LOC544267)/Cystathionine beta-synthase	Solyc08g014340.3.1	0.94	3.29E-03	Amino acid metabolism	Biosynthesis of suphur containing amino acids
**Les.4693**	Pathogenesis-related protein P4(P4)	Solyc09g007010.1.1	0.94	4.15E-03	Plant defense	Plant defense against pathogens and stress response
**LesAffx.62690**	Cytokinin riboside 5'-monophosphate phosphoribohydrolase LOG8(LOC101252798)	Solyc08g062820.3.1	0.938	2.16E-04	Plant Growth and Development	Cytokinin metabolism
**LesAffx.22812**	probable E3 ubiquitin-protein ligase RNF217(LOC101250202)	Solyc03g117860.3.1	0.931	8.27E-04	Protein Metabolism	RING finger family of E3 ubiquitin ligases involved in protein-turnover
**Les.3034**	Wound-induced proteinase inhibitor 2(LOC101255652)	Solyc11g020960.2.1	0.927	4.37E-04	Plant Defense	Plant defense against herbivores and pathogens
**LesAffx.4763**	MADS-box transcription factor(LOC101247413)	Solyc12g087830.2.1	0.92	7.32E-03	Plant Growth and Development	Flower development, fruit development, and ripening
**Les.2909**	Phosphoenolpyruvate carboxylase(PEPC2)	Solyc07g055060.3.1	0.912	1.37E-03	Plant Growth and Development	Photosynthetic Carbon fixation
**LesAffx.17150**	Nitrite reductase(Nii1)	Solyc01g108630.3.1	0.898	6.89E-03	Plant Growth and Development	Nitrogen Metabolism
**Les.3700.1**	Non-symbiotic hemoglobin class 1(Glb1)	Solyc07g008240.3.1	0.868	2.33E-03	Cell Metabolism and Stress Response	nitrate assimilation, carbon metabolism, and the plant defense against oxidative stress
**Les.3539**	Phosphoenolpyruvate carboxylase kinase 1(PPCK1)	Solyc04g009900.4.1	0.801	6.58E-03	Plant Growth and Development	Photosynthetic Carbon fixation

We also adjusted the raw data *p*-value (*p*_calcualted_-value) using the conservative Bonferroni correction method at *p*-value cut-off of 0.05 (*P*_bonferroni_-value < 0.05) to retrieve the significant genes, and the results were compared among experimentally derived raw data *p*-value (*P*_calculated_-value) at the various P_value_ thresholds (*p*_cal_ < 0.05, *p*_cal_ < 0.01, and the *p*_cal_ < 0.001), and with the FDR corrected Benzamanii Hochberg (*P*_adjusted_-value < 0.05) to find the significant genes. Interstingly, we found only 07 genes common between experimentally derived raw data *p*-value, FDR corrected *P*_adjusted_ -value (*P*_adj_-value < 0.05), and (*P*_bonferroni-_value < 0.05). In one report, Slonim [100] pursued the distinction of genes between BRCA1 and BRCA2-mutation-positive tumors, using microarrays and computing a modified F statistic. A *p*-value threshold of 0.001 revealed 51 significant genes out of 3,226, with approximately three expected false positives. By lowering the threshold to 0.0001, their subsequent analysis indicated 9–11 differentially expressed genes, deepening insights into tumor-specific gene expression variations. Similarly, in order to find the legitimate DNA–protein binding and reducing the false positive results [101] evaluated the binding of 106 transcription factors across the genome in yeast and the binding was measured based on *p*-value under the null hypothesis that no binding occurs, resulting in the consideration of thousands of *p* values. Interstingly, 3,985 interactions found to be significant at this threshold, ≈ 6–10% are false positives were reported at a threshold *p*-value of 0.001 which could be explained by the fact that at this threshold, we could have a maximum inclusion of legitimate regulator–DNA interactions with minimum false positives which further support our results. In fact, presence of the common genes across the two different statistical methods holds an important implication for the robustness of the our findings and this overlapping suggested a convergence of evidence from two different statistical methods, indicating a higher level of confidence in the observed changes in gene expression. For example, Anders et al. [102] reported the genes that are found to be differentially and significantly expressed both at the FDR adjusted *p*-value (q-value) and/or raw *p*-value (*p*_calculated_-value) will show a strong degree of confidence in the results which further supports our findings Overall, the DEGs bioregulation analysis uncovered the relevance of the statistical parameters in understanding the gene-regulatory dynamics. Our results identified the common and most significant set of genes that were specifically involved in metabolic and biochemical alteration in the host(tomato) tissues under the effect of *Trichoderma* T34 induced microbial priming and its interaction with host tissues during a late colonization event. The presence of common genes at stringent *p*-value cut-off scores across all the different probability thresholds highlights their critical function in regulating the transcriptional network and signaling cascades during *Trichoderma*-tomato interaction.

### Transcriptomic characterization and identification of the DEGs

Out of those 156 common transcripts that were found to be common at all the probability threshols values (*p*_cal_-value < 0.05, *p*_cal_-value < 0.01, and *p*_adj-_value < 0.05), the topmost upregulated hits based on fold changes (FC) values with FC > 1 included an uncharacterized PD-(D/E)XK superfamily protein (LOC101251740) (Solyc02g078150.4), Neryl diphosphate synthase 1(CPT1), Threonine dehydratase biosynthetic (chloroplastic) (TD2) (Solyc09g008670.3), BAHD acyltransferase DCR (Solyc05g052670.1), Organ-specific protein S2 (Solyc10g009150.3), probable Galacturonosyltransferase-like 1 (Solyc04g079860.1), Lipoxygenase (loxC) (Solyc01g006540.4), Pyruvate kinase 1 (cytosolic) (Solyc09g008840.4), Protein early flowering 2-like (Solyc04g064870.3). Furthermore, the topmost downregulated hits with FC < 1 reported were Non-symbiotic hemoglobin class 1(Glb1) (Solyc07g008240.3), Ferredoxin–nitrite reductase (chloroplastic) (Solyc01g108630.3), Phosphoenolpyruvate carboxylase (PEPC2)(Solyc07g055060.3), Phosphoenolpyruvate carboxylase kinase 1(PPCK1) (Solyc04g009900.4), Pathogenesis-related protein P4 (Solyc09g007010.1), Wound-induced proteinase inhibitor 2(Solyc11g020960.2), Cytokinin riboside 5'-monophosphate phosphoribohydrolase LOG8(Solyc08g062820.3), UDP-glycosyltransferase 76E1 (UGT76E1) (Solyc10g085230.2), MADS-box transcription factor (Solyc12g087830.2), Adenylyl-sulfate reductase (Solyc02g080640.4). When we compared the significant genes based on FDR corrected *p*_adj_-value < 0.05 and *p*_adj_-value < 0.01 with *p*_cal_-value < 0.05 and *p*_cal_-value < 0.01, we reported 51 common and significant genes between *p*_adj-value_ < 0.05; *p*_adj_-value < 0.01; *p*_cal_-value < 0.05, and *p*_cal_-value < 0.01). However, based on the FDR corrected *p*_adj_-value and *p*_cal_-value, the most significant list of common genes(among the two p-value thresholds) reported that were differentially expressed (including both upregulated and the downregulated) in un-inoculated control vs *Trichoderma* T34 primed tomato plants were Neryl diphosphate synthase 1(CPT1), UDP-glycosyltransferase 76E1(UGT76E1), Lipoxygenase (loxC), Threonine dehydratase biosynthetic (chloroplastic) (TD2), S-adenosylmethionine decarboxylase 2(LOC101260400), Aspartic protease inhibitor 1(LOC101262903), E3 ubiquitin-protein ligase RNF217(LOC101250202), Non-symbiotic hemoglobin class 1(Glb1), Sesquiterpene synthase 1(SSTLE1), Protein PIN-LIKES 3-like (LOC101247117), Pyruvate kinase 1, cytosolic (LOC101248036), Transcription factor MYB13-like (LOC101260654), Adenylyl-sulfate reductase (LOC544267), Phosphoenolpyruvate carboxylase (PEPC2), Chitinase (CHI3), Phosphoenolpyruvate carboxylase (PEPC2), Phosphoenolpyruvate carboxylase kinase 1(PPCK1), Pathogenesis-related protein P4(P4), Formate dehydrogenase(FDH), Cysteine proteinase 3(Cyp-3), Lactoylglutathione lyase(LOC101251435), Putative methyltransferase DDB_G0268948(LOC101256176),Pprotein PIN-LIKES 3-like (LOC101247117), Auxin repressed/dormancy associated protein (LOC101258429), *cis*-Prenyltransferase 7. Since we encountered 155 common DEGs at *p*_cal_-value < 0.05 and *p*_adj_-value < 0.05, and 156 common transcripts differentially expressed in both *p*_adj_-value < 0.05 and *p*_cal-value_ < 0.01, the topmost hits (both upregulated and downregulated) found with stringent cut-off scores (without FDR correction) at *p*_cal_-value < 0.01 and the significant genes have been reported (Table 1).

Interestingly, a comparative transcriptional profiling at two different *p*-values (*p*_adj_-value < 0.05 and *p*_cal-_value < 0.01) revealed the presence of all the significant genes that were also present at the *p*_cal-_value < 0.01 that were obtained at the *p*_adj-_value < 0.05). Overall, at a lower stringent cut-off *p*_cal_-value < 0.01, we found 491 genes expressed across differentialy across the tomato genome under the two cross-comparable groups with 289 genes upregulated (FC > 1) at both *p*_cal_-values (*p*_cal_ < 0.05 and *p*_cal_ < 0.01) and 202 genes downregulated (FC < 1) (Fig. 4d). Some of the transcripts found at *p*_cal_-value < 0.01 and FC > 1.1 included Lipoxygenase (LoxC) (Solyc01g006540.4), Sesquiterpene synthase 1(SSTLE1) (Solyc06g059930.4),PD-(D/E)XK nuclease superfamily uncharacterized protein (Solyc02g078150.4.1) playing a critical role in addressing multiple nucleic acid maintenance issues, Pyruvate kinase 1, cytosolic (Solyc09g008840.4.1), threonine dehydratase biosynthetic (chloroplastic)(TD2) (Solyc09g008670.3), BAHD acyltransferase DCR(Solyc05g052670.1), Neryl diphosphate synthase 1(CPT1) (Solyc08g005680.4), Bet v I/Major latex protein domain-containing protein (Kirola) (Solyc10g048030.2), Formate dehydrogenase(FDH) (mitochondrial) (Solyc02g086880.4.1), Protein early flowering 2-like (Solyc04g064870.3), Organ-specific protein S2(Solyc10g009150.3), Galacturonosyltransferase-like 1(Solyc04g079860.1), and plant cell wall protein SlTFR88(LOC778266). Furthermore, in the down-regulated section (*p*_cal_-value < 0.01) and FC > 0.95 some of the transcripts reported were non-symbiotic hemoglobin class 1(Glb1) (Solyc07g008240.3.1), protein REVEILLE 1 (Solyc02g036370.3), Ferredoxin–nitrite reductase (chloroplastic) (Solyc01g108630.3), Phosphoenolpyruvate carboxylase (PEPC2) (Solyc07g055060.3), Phosphoenolpyruvate carboxylase kinase 1(PPCK1) (Solyc04g009900.4), G-type lectin S-receptor-like serine/threonine-protein kinase SD3-1(Solyc03g063650.1), Pathogenesis-related protein P4 (Solyc09g007010.1), Probable E3 ubiquitin-protein ligase RNF217(Solyc03g117860.3), wound-induced proteinase inhibitor 2(Solyc11g020960.2), Cytokinin riboside 5'-monophosphate phosphoribohydrolase LOG8(Solyc08g062820.3), ultraviolet-B receptor UVR8 isoform X1 (Solyc05g052950.4.1), UDP-glycosyltransferase 76E1 (UGT76E1) (Solyc10g085230.2), MADS-box transcription factor (Solyc12g087830.2), Adenylyl-sulfate reductase (Solyc02g080640.4). Furthermore, presence of common transcripts in both FDR corrected (*p*_adj -_value < 0.05) and calculated probability value (*p*_calculated_-value) at three different cut-off scores (*p*_cal_ < 0.05, *p*_cal_ < 0.01, and *p*_cal_ < 0.001) revealed the significance of calculated *p*-value in finding the significant transcripts. The presence of 22 DEGs in all the probability groups (*p*_cal_-value < 0.05, *p*_cal_-value < 0.01, *p*_cal_ < 0.001, and *p*_adj_ < 0.05) revealed the most significant DEGs that are differentially expressed across the two contrasting groups (un-inoculated control vs *Trichoderma*-tomato) and includes Neryl diphosphate synthase 1(CPT1), E3 ubiquitin-protein ligase RNF217(LOC101250202), Cytokinin riboside 5'-monophosphate phosphoribohydrolase LOG8(LOC101252798), N-acetyl-glutamate synthase(LOC100301981), sesquiterpene synthase 1(SSTLE1), G-type lectin S-receptor-like Serine/threonine-protein kinase SD3-1(LOC101250670), Glyceraldehyde 3-phosphate dehydrogenase(GAPDH), S-Adenosylmethionine decarboxylase 2(LOC101260400), Isopentenyl diphosphate isomerase(IDI1), Wound-induced proteinase inhibitor 2(LOC101255652), RNA pseudouridine synthase A 1(LOC101244488), Mitochondrial succinate-fumarate transporter 1(LOC101266282), Polyol transporter 5-like(PMT5), tRNA Pseudouridine synthase A 1(LOC101244488), protein ETHYLENE-INSENSITIVE 3-like 4(EIL4), and kirola (LOC101264616). Moreover, cross comparsion of the DEGs based on experimentally derived raw data *p*-values including *P*_cal_ < 0.05, *P*_cal_ < 0.01, *P*_cal_ < 0.001 and FDR corrected *P*_adjusted_ < 0.05 and even at more stringent *P*_adjusted_ < 0.01 values revealed 13 significant genes with differential expression including neryl diphosphate synthase 1(CPT1), sesquiterpene synthase 1(SSTLE1), wound-induced proteinase inhibitor 2(LOC101255652), Cytokinin riboside 5'-monophosphate phosphoribohydrolase LOG8(LOC101252798) S-Adenosylmethionine decarboxylase 2(LOC101260400), Organ-specific protein S2(LOC101245207), probable E3 ubiquitin-protein ligase RNF217(LOC101250202), Glutaminase domain-containing protein(LOC544312), protein RICE SALT SENSITIVE 3(LOC101258771), Uncharacterized LOC101251740 (LOC101251740), and Kirola (LOC101264616). The full table of the differentially expressed transcritpts across the two cross-comparable groups has been shown in Table 1.

Overall, transcriptomic profiling for identification and characterization of the differentially expressed genes (DEGs) with significant fold change values during *Trichoderm*-tomato interaction at post colonization events revealed that the identified and characterized DEGs were associated with functional categories like secondary metabolism (biosynthesis of plant hormones, pigments, isoprenoids, flavonoids, monoterpenes, chalcones), regulation of RUBISCO activity and optimizing photosynthesis, DNA and RNA metabolism, biosynthesis of protein, and amino acids, protein folding, and chaperone activity, Nitrogen metabolism, and assimilation, carbohydrate metabolism, biosynthesis and re-inforcement of cellular structure, phytohormone biosynthesis, and metabolism, fruit ripening and flavour enhancement, plant growth, development, and improving productivity, alleviation of abiotic and biotic stress along with regulation of the programmed cell death.

### Differential expression of PR proteins

The differential expression of various PR genes including PR 2 (β-1,3-glucanases), PR 3 (chitinases), PR 4 (antifungal) PR 5, Thaumatin, Defensin and the Thionins was also reported. Based on protein sequences we searched the total expressed sequence tags (ESTs) available across the tomato genome with specific protein sequence and the collected ESTs were sorted from the list of total DEGs. The results analyzed at *p*_adj_-value < 0.05, *p*_cal_-value < 0.01 and *p*_adj_-value < 0.05 reported the common expression of acidic endochitinase 26 KDa (Solyc02g082920.4) in all the probability groups with downregulated expression (FC < 1). Further, two other acidic endochitinases including endochitinase 27 kDa (Solyc02g082930.3) and other Solyc05g050130.4.1(acidic endochitinase) was reported to be common in *p*_cal_-value < 0.05 and *p*_adj_-value < 0.05 but not in *p*_cal_-value < 0.01. Nevertheless, 30 KDa basic endochitinase (Solyc10g055810.2.1) was present in all the probability groups including *p*_cal_ < 0.05, *p*_cal_ < 0.01, and *p*_adj_ < 0.05 and had upregulated expression(FC > 1). In contrast, we found one more plant specific chitinase ClassV chitinase (LOC101257483) common in *p*_cal_-value < 0.05, *p*_cal_-value < 0.01 and even at *p*_cal_-value < 0.001 but not in *p*_adj_-value < 0.05 was found to be upregulated (FC > 1). In fact, Class V chitinases are plant-specific chitinases that are characterized by a conserved cysteine-rich domain and a chitin-binding domain and reported to play a crucial role in plant defense against phytopathogens and also involved in various developmental processes. Furthermore, differential expression of two β-1,3-glucanases (PR-2) proteins including Solyc01g008620.4 and Solyc01g060020.4 was reported to present in *p*_adj_-value < 0.05 but not in *p*_cal_ < 0.05 and *p*_cal_ < 0.01. The isoform of PR2 (Solyc01g060020.4) was found to have upregulated expression profile (FC > 1) compare to the other isoform Solyc01g008620.4 with down-regulated expression profile (FC < 1). However, differential expression of other isoform of PR 2 protein Solyc01g080220.3 was present in both *p*_cal_ < 0.05 and *p*_cal_ < 0.01 but not in the *p*_adj_ < 0.05 Table 3. Apart from PR 2 and PR 3, the other PR genes including Barwin-domain proteins (PR 4), and thaumatin-like (PR 5) was also reported. Interstingly, both PR 4 (Solyc09g007010.1) and PR5 (Solyc08g080670.1) was reported in both *p*_adj-_value < 0.05 and *p*_cal-_value < 0.05. In contrast, P4 was present in all the probability values like *p*_adj-_value < 0.05, *p*_cal-_value < 0.05 and *p*_cal_-value < 0.01. Wheras the PR4 protein was downregulated (FC < 1), the PR5 was upregulated (FC > 1). These results clearly indicate the relevance of the *p*-value in selecting and deciding the statistically significant transcripts that was differentially expressed across the tomato genome under the two contrasting experimental groups. The heat map showing the differential expression of all the PR proteins, transcription factors at all the probability groups (*p*_cal_-value < 0.05; *p*_cal_-value < 0.01; *p*_adj_-value < 0.05) (Fig. 3c).

**Table 3:** Table showing the differential expression of the PR genes and various defense-related transcription factors expressed differentially during the *Trichoderma*-tomato interaction (T1, T2, and T3) vs un-inoculated control (C1, C2, and C3). The significant genes have been sorted based on *p*_cal_-value < 0.05. The identified and characterized proteins were further validated using the Sol Genomics BLASTp tool to find the first and topmost hit related to the specific isoform of the protein

**Probe ID**	**Putative Identity**	**Sol Genomics ID**	**Fold Change**	***p*-value**	**Log 2 FC**	**Putative function**	**References**
**Les.122**	Acidic 26-kDa endochitinase	Solyc04g009900.4.1	0.955	6.00E-03	-6.61E-02	Chitinolytic antifungal activities	[103, 104]
**Les.3406**	Basic 30-kDa endochitinase	Solyc10g055810.2.1	1.032	2.82E-03	4.49E-02	Chitin degradation, plant defense against fungal pathogens	[103, 104]
**Les.3779**	Acidic 27-kDa endochitinase	Solyc02g082930.3.1	1.053	1.32E-02	7.50E-02	acidic 27-kDa endochitinase cleaves the β-1,4 glycosidic bonds in chitin,	[103, 104]
**Les.2591**	glucan endo-1,3-beta-glucosidase B precursor	Solyc01g060020.4.1	1.04	1.45E-02	5.60E-02	Plant defense through hydrolytic clevage of β (1 → 3)-glucosidic linkages in β (1 → 3)-d-glucan, present in fungal cell walls	[104–106]
**Les.3673**	Beta-1,3 Glucanase	Solyc01g008620.4.1	0.848	3.39E-02	-2.37E-01	Plant defense through hydrolytic clevage of β (1 → 3)-glucosidic linkages in β (1 → 3)-d-glucan, present in fungal cell walls	[104–106]
**Les.4307**	Pathogenesis-related 5(PR-5)/thaumatin-like proteins (TLPs)	Solyc08g080670.1.1	1.132	2.77E-02	1.79E-01	Plant Defense	[104]
**Les.4693**	Pathogenesis-related 4(PR-4)	Solyc09g007010.1.1	0.94	4.15E-03	-8.94E-02	Plant Defense against fungal pathogen	[106]
**Les.4460**	Pathogenesis-related precursor 2(PR2)	Solyc01g097240.3.1	0.977	4.08E-02	-3.35E-02	Plant Defense	[107]
**LesAffx.5691**	Pathogenesis-related 4(PR-4)	Solyc09g006005.1.1	1.06	1.83E-02	8.46E-02	Plant Defense	[106]
**Les.4496.1**	Pathogenesis-related protein STH-2-like (PRSTH2)	Solyc09g090970.4.1	1.039	8.79E-03	5.52E-02	PR-10 like protein provide defense against abiotic and biotic stress	[104]
**LesAffx.4763**	MADS-box transcription factor(LOC101247413)	Solyc12g087830.2.1	0.92	7.32E-03	-1.20E-01	floral transition, floral patterning, and other reproductive development	[104–106, 108, 109]
**Les.4450.1**	MADS-box transcription factor RIN(RIN)	Solyc05g012020.4.1	0.956	2.14E-02	-6.53E-02	Key regulator of fruit ripening gene-expression network	[110, 111]
**Les.3716.1**	SANT/MYB domain protein	Solyc10g052470.1.1	0.958	1.01E-02	-6.23E-02	Early stages of fruit development	[112]
**Les.5091.1**	MYB4 transcription factor	Solyc09g090130.3.1	1.031	4.78E-03	4.38E-02	Plant growth and Development and tolerance to various abiotic stresses	[113, 114]
**LesAffx.65724**	transcription factor MYB20(LOC101252939)	Solyc02g093730.4.1	1.021	2.96E-02	3.01E-02	Regulating the phenylpropanoid metabolism during secondary cell wall formation	[115]
**LesAffx.53591**	Transcription factor MYB13-like(LOC101260654)	Solyc06g083900.3.1	1.081	1.82E-03	1.13E-01	Auxin response and flavonoid biosynthesis	[116, 117]
**LesAffx.62138**	Transcription factor MYB78-like(LOC101265734)	Solyc05g053330.3.1	0.975	2.74E-02	-3.68E-02	Abscission Zone-Specific Modulation of Key Meristem Activity Genes	[118]
**LesAffx.21940**	MYB family transcription factor (LOC101243935)	Solyc01g108300.3.1	1.03	1.58E-02	4.23E-02	Plant growth and Development	[118]
**Les.3512.1**	WRKY transcription factor 2(WRKY2)	Solyc07g066220.3.1	0.988	1.05E-02	-1.79E-02	Abiotic and Biotic Response	[119, 120]
**LesAffx.9910**	Probable WRKY transcription factor 40 (LOC101246812)	Solyc03g116890.3.1	0.988	1.86E-02	-1.80E-02	Biotic Response	[119, 121–124]
**Les.3964.1**	Probable WRKY transcription factor 17 (LOC543855)	Solyc12g096350.2.1	0.952	1.72E-02	-7.04E-02	Negative regulator of basal resistance in Arabidopsis	[125]
**Les.5219.1**	NAC domain-containing protein NACMTF3(NACMTF3)	Solyc06g073050.2.1	0.99	1.45E-02	-1.45E-02	Biotic interaction	[126]

### Characterization of transcriptional regulatory network for systemic defense and flowering

**Fig. 5:**
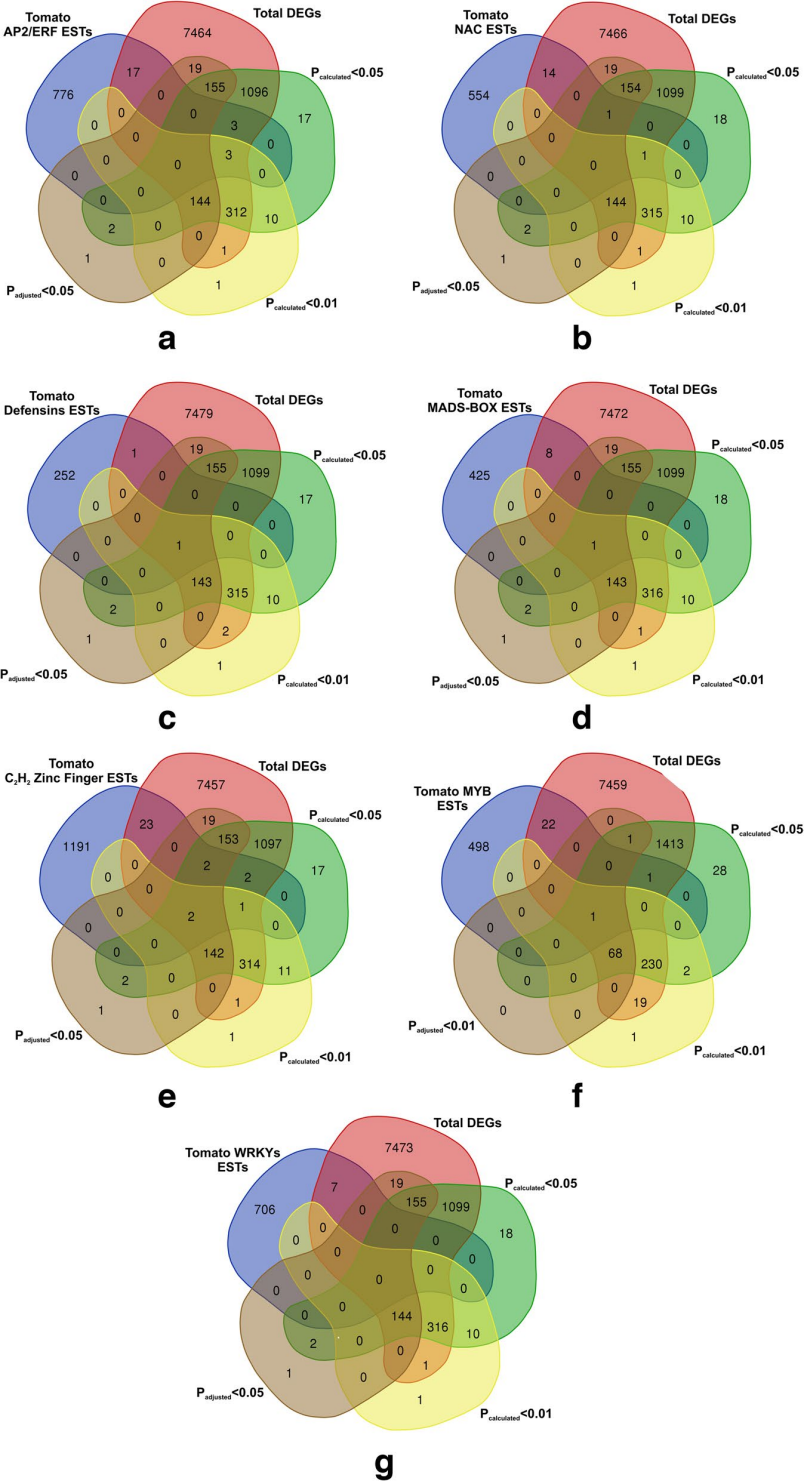
The Vein digrammes showing the presence of common and shared gene-specific family related transcription factors. The DEGs were sorted based on FDR corrected and raw data based experimentally derived (Ttest) based calculated *p*-values. We compared the DEGs specific to gene-families with the maximum and total possible ESTs specific and related to a particular gene families. The ESTs were sorted based on gene-specific family related function domain and were compared with the DEGs sorted based on (*P*_cal_ < 0.05, *P*_cal_ < 0.01, *P*_adj_ < 0.05). **a** The Vein digramme sorting the AP2/ERF family specific ESTs available across the tomato genome and their filtering from the significant DEGs (*P*_cal_ < 0.05, *p*_cal_ < 0.01, and *P*_adj_ < 0.05). **b** The Vein digramme sorting the NAC family specific ESTs available across the tomato genome and their filtering from the significant DEGs (*P*_cal_ < 0.05 and *P*_adj_ < 0.05). **c** The Vein digramme sorting the Plant defensins family specific ESTs available across the tomato genome and their filtering from the significant DEGs (*P*_cal_ < 0.05, *P*_cal_ < 0.01, and *P*_adj_ < 0.05). **d** The Vein digramme sorting the MADS box family specific ESTs available across the tomato genome and their filtering from the significant DEGs (*P*_cal_ < 0.05, *P*_cal_ < 0.01, *P*_adj_ < 0.05, and *P*_adj_ < 0.01). **e** Vein digramme showing the C_2_H_2_ Zinc Finger family. **f** The Vein digramme showing the MYB members. **g** The Vein digramme showing the WRKY members

*Trichoderma* spp. enhances plant defenses through priming, activating the MAPKs and key transcription factors (WRKYs, MYBs, MYCs) in response to stress, resulting in faster and stronger immune responses. These transcription factors are pivotal in priming, serving as key regulators in the transcriptional network for systemic defense following stress recognition. Based on presence of specific functional domains/proteins we searched and retrieved all the possible/available ESTs (associated with specific functional domain or representing a specific gene-family) across the tomato genome and compared the data with *T.harzianum*-tomato expression data (GSE76332) for finding the common transcripts that were differentially expressed during the *Trichoderma*-tomato interaction in between the un-incoculated host as control vs host incoculated with *Trichoderma* as treatment (two contrasting groups). *T. harzianum* T34 priming resulted into differential expression of transcriptional factors related to plant growth and development, flowering and systemic defense. We identified and characterized 17AP2/ERF accessions/hits that were expressed across the tomato genome during microbial priming with *T. harzianum* T34. However, only six significant ESTs were reported to be differentially expressed (*P*_cal_ < 0.05), of which, three members were reported to be differentially expressed at more significant and lower probability cut-off (*P*_cal_ < 0.01). In this way, we reported three common AP2/ERF members (ERF5, ERF3 and the ERF1B) that were differentially expressed under the defined experimental conditions across the two cross-comparable experimental groups (Fig. 5a). Interestingly, genome-wide transcriptomic profiling during *Trichoderma*-tomato interaction revealed differential expression of only one NAC member NP_001234482.1(Solyc04g009440.3) and was found to be differentially expressed and downregulated at *p*_adj_ < 0.05 and *p*_cal_-value < 0.05 but not at the *p*_cal_-value < 0.01 (Fig. 5b). Among the plant defensin family, we reported defensin like protein precursor (NP_001333453; Solyc07g007755.1) differentially expressed (significant) and was commonly expressed (*P*_adj_ < 0.05, *P*_cal_ < 0.05, and *P*_cal_ < 0.01) (Fig. 5c). The full length gene prediction for this defensin like protein precursor identified and characterized it to be a "novel tomato I-3 gene" showing resistance against the Fusarium wilt. Likewise, we found only one MADS box member (NP_001352612.1; Solyc12g087830.2) was commonly expressed at different cut-off probability cut-off values (*P*_cal_ < 0.05, *P*_cal_ < 0.01, and *P*_adj_ < 0.05) and even at more stringent FDR corrected p_adjusted_ cut-off values (*P*_adj_ < 0.01) and was reported to be downregulated under the *T. harzianum* treatment conditions (*p*_cal_-value; 7.32E-03; Log2 FC, -1.20E-01). The presence of this EST at both *p*_adjusted_-values (FDR corrected) and *P*_calculated_-value confirms the role of MADS box transcription factor (Solyc12g087830.2) during *Trichoderma*-tomato interaction (Fig. 5d). In the C_2_H_2_ Zinc finger family, we reported genome-wide expression of 29 ESTs associated with this family. However, four EST accessions found that were differentially expressed and common at both *P*_adj_ < 0.05 and *P*_cal_ < 0.05 cut-off scores were Zinc finger 3 (Solyc06g068390.1), Zinc Finger protein ZAT 10 like (Solyc12g088390.1.1), Polyol transporter 5 like (Solyc01g109460.3.1) and A20/AN1 zinc finger protein (Solyc01g014180.3.1) were found (Fig. 5e). Interstingly, we also found A20/AN1 zinc finger protein (Solyc01g014180.3.1) and Polyol transporter at lower *p*-value of *p*_cal_ < 0.01. Nevertheless, only one EST aceession of the zinc finger family, the Polyol transporter 5 like (Solyc01g109460.3.1) was found at *P*_cal_ < 0.05, *P*_cal_ < 0.01, *P*_adj_ < 0.05, and *P*_cal_ < 0.001 showing the significance of this protein during plant-fungus interaction. For the MYB family in tomato, we encountered genome-wide differential expression of 22 transcription factors. However, based on different p cut-off score values, we found only two MYB accessions MYB 13 (XP_004242359.1; Solyc06g083900.3) and transcription factor MYB78 proteins (*P*_cal_ < 0.05). Interstingly, out of total differentially expressed 22 MYB transcripts, we reported only 1 member that was differentially expressed across the tomato genome at all the stringent p-cut-off score values including *p*_cal_ < 0.05, *p*_cal_ < 0.01, *p*_adj_ < 0.01, and even at *P*_adj_ < 0.01 (Fig. 5f). Interstingly, for *WRKY* gene family, we did not encounter any significant members or EST accessions at any p-value cut-off and all the reported members were found to be non-significant (Fig. 5g). The list of all the differentially expressed and significant transcription factors along with their putative fuction and *p*-value cut-off scores has been shown in Table 4. Overall, based on our results, we reported that during *Trichoderma*-tomato interaction resulted into differential and specific expression of transcription factors involved in regulating the transcriptional network playing a critical role in plant growth and development, flowering, and systemic defense.

**Table 4:** List of gene-specific family related transcription factors that were differentially expressed and involved in regulating the transcriptional regulatory network for systemic defense, plant growth and flowering in tomato during microbial priming with *T. harzianum* T34. We have shown significant transcription (*p*-value) factors along with some of the non-significant genes (*P*_cal_ > 0.05) that were reported to be differentially expressesed at *P*_cal_ < 0.05 (significant). Some of the reported transcription factor were common to the *P*_adj_ < 0.05 and *P*_cal_ < 0.01 and even more stringent cut-off scores like *P*_adj_ < 0.01

**S. No**	**Protein Name**	**EST accession identity**	**Probe ID**	**NCBI Accession**	***p*-value**	**Log 2FC**	**Function**	**Significance**
**ERF Family**								
**1**	Ethylene responsive transcription factor WIN1/SHN1	BG642554	LesAffx.19017	XP_004235965.1	8.38E-01	9.39E-03	Cutin Biosynthesis, Multiple Abiotic stress Tolerance	Non-significant (*P*_cal_ > 0.05)
**2**	Ethylene-responsive transcription factor SHINE3	AW928465	LesAffx.41596	NP_001306131.1	1.32E-01	6.76E-02	Biosynthesis of Cutin and ensuring proper floral organ morphology and surface formation	Non-significant (*P*_cal_ > 0.05)
**3**	Ethylene-responsive transcription factor ABR1-like	AI484721	LesAffx.63587	XP_004237483.1	2.39E-01	5.50E-03	APETALA2-Domain Transcription Factor Functions as a Repressor of ABA Response	Non-significant (*P*_cal_ > 0.05)
**4**	Ethylene-responsive transcription factor ERF021	AI771296	LesAffx.64452.1	XP_004250714.1	1.98E-02	-6.91E-02	Unknown	Significant (*P*_cal_ < 0.05)
**5**	Ethylene-responsive transcription factor ERF16	AI898830	LesAffx.71529.1	XP_004241075.1	3.74E-02	-1.69E-02	Unknown	Significant (*P*_cal_ < 0.05)
**6**	Ethylene-responsive transcription factor ERF2	AY192368	Les.4102.1	AAO34704.1	3.07E-02	-2.37E-02	Seed Germination Salt Tolerance	Significant (*P*_cal_ < 0.05)
**7**	Ethylene-responsive transcription factor ERF5	AY559315	Les.4531.1	NP_001317374.2	4.44E-03	-2.09E-02	Biotic Stress Tolerance SA and JA signaling	Significant (*P*_cal_ < 0.05, *P*_cal_ < 0.01)
**8**	Ethylene-responsive transcription factor ERF3	AW222053	LesAffx.41457.1	XP_004243505.1	8.63E-03	-4.08E-02	Abiotic Stress Tolerance	Significant (*P*_cal_ < 0.05, *P*_cal_ < 0.01)
**9**	Ethylene-responsive transcription factor 1B	BG627344	Les.876.1. A1_at	XP_004236685.1	6.63E-03	2.01E-02	Negative regulator of osmotic resistance	Significant (*P*_cal_ < 0.05, *P*_cal_ < 0.01)
**NAC Family**								
**1**	NAC domain protein	AY498713	Les.4483.1	NP_001234482.1	1.22E-02	-7.34E-02	ET biosynthesis and Fruit ripening	Significant
**2**	NAC transcription factor NOR (No-ripening)	BM410927	Les.288.1	NP_001234652.1	1.70E-01	-4.09E-02	positive regulator of fruit ripening	Non-Significant
**MADS Box Family**								
**1**	MADS-box transcription factor	BG123322	LesAffx.4763.3	NP_001352612.1	7.32E-03	-1.20E-01	Flowering	Significant (*P*_cal_ < 0.05, *P*_cal_ < 0.01, *P*_adj_ < 0.05, and *P*_adj_ < 0.01
**2**	MADS-box protein JOINTLESS	AI895411	LesAffx.71484.1	XP_010312646.1	6.05E-01	3.62E-02	Tomato flower abscission zone development	Non-Significant *P*_cal_ > 0.05
**3**	floral homeotic protein PISTILLATA-like/APETALA3 (AP3)	BE354620	Les.2902.1	NP_001234075.2	2.87E-01	4.44E-02	Flowering	Non-Significant *P*_cal_ > 0.05
**Plant Defensins Family**								
**1**	Defensin like Protein Precursor	BG127217	Les.4596.1	NP_001333453.1	8.26E-03	4.15E-02	Disease Resistance (Fusarium wilt I3 introgressed resistance)	Significant (*P*_adj_ < 0.05, *P*_cal_ < 0.01, and *P*_cal_ < 0.05)
**C**_**2**_**H**_**2**_** Zinc Finger Family**								
**1**	Zinc Finger Protein 3	BG626790	Les.661.1.A1_	XP_004241683.1	4.73E-02	-1.12E-01	Abiotic and Biotic Stress Tolerance	Significant (*P*_adj_ < 0.05 and *P*_cal_ < 0.05)
**2**	Zinc Finger Protein ZAT10 like	AW034622	LesAffx.36193.1	XP_004252818.1	2.64E-02	1.27E-01	Transcription repressor under abiotic stresses;	Significant (*P*_adj_ < 0.05 and *P*_cal_ < 0.05)
**3**	Polyol Transporter 5 like	AW041670	LesAffx.10299.1	NP_001287655.1	5.42E-05	8.91E-04	Sugar Transporter	Significant (*P*_adj_ < 0.05, *P*_cal_ < 0.01, *P*_cal_ < 0.001, and *P*_cal_ < 0.05)
**4**	A20/AN1 zinc finger protein	AW218130	Les.2416.1	NP_001307087.1	-5.59E-04	7.65E-03	Stress-associated protein	Significant (*P*_adj_ < 0.05, *P*_cal_ < 0.05, *P*_cal_ < 0.01)
**MYB Gene Family**								
**1**	MYB13 like	AI899018	LesAffx.53591.1	XP_004242359.1	1.82E-03	1.13E-01	Leaf Development	Significant *p*_cal_ < 0.05, *p*_cal_ < 0.01, *p*_adj_ < 0.01, and _*P*adj_ < 0.01
**2**	MYB78	AW737374	LesAffx.62138.1	XP_004239882.1	2.74E-02	-3.68E-02	Abscission zone-specific	Significant *P*_cal_ < 0.05
**WRKY Gene Family**								
**1**	WRKY transcription factor IId-1	AW221937	LesAffx.735.1	NP_001308545.1	1.64E-01	4.44E-02	Biotic and Abiotic Stress	Non-Significant
**2**	WRKY transcription factor 31	AW621251	Les.2667.2	NP_001306910.1	5.70E-01	9.88E-03	Biotic Stress	Non-Significant

### Gene prediction

**Fig. 6:**
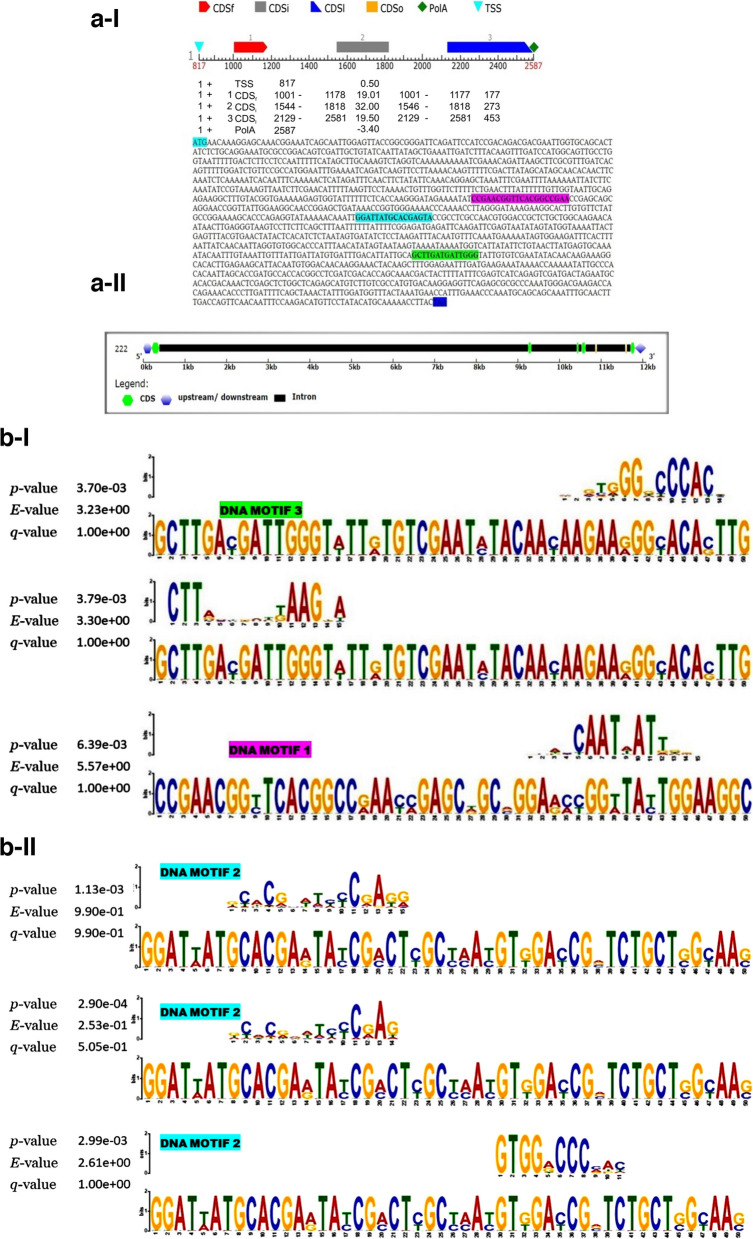
Gene prediction, and structural organization of the gene specific transcription factors that were reported to be differentially expressed showing the position of transcriptional start site (TSS), coding sequence (CDS), and polyadenylation site (poly A tail). **a-I** Soft berry gene prediction showing the position of NAC transcription factor **a-II**. Gene structure display server showing the structure of gene consisting of introns, exons, and intron–exon boundaries. DNA motif analysis showing the putative motifs and their alignment with the motifs present in database. The DNA motifs alignment with query read motifs showing the functional regulatory network asscoaited with the specific sequences. Tomtom is a motif comparison algorithm that ranks the target motifs in a given database according to the estimated statistical significance of the match between the query and the target.The putative DNA motifs associated with NAC transcriptional factor based on sequence alignment of the query reads with sequence reads available for using *Arabidopsis* JASPAR database **b**-**1** and **II**

Gene prediction was done to identify and characterize the genomic locus of the identified transcription factors. The genomic locus of the NAC transcription factor with respect to transcriptional start site, coding sequences, poly-adenylation tail (poly A) tail was retrieved (Fig. 6a-I). Gene prediction results for NAC protein showed that it consist of 3 exons with intron–exon boundaries. The genomic locus of the MADS box transcription factor across the tomato genome with respect to transcriptional start site (TSS), coding sequences (CDS) and polyadenylation tail (Poly A) tail. Gene prediction of the identified and characterized MADS-Box transcription factor consisted of seven exons and six introns (Fig. 6a-II). The position of the exons across the tomato genome was reported as > NC_015449.3: 3:64306,262–64306446, 64315308–64315386, 64316475–64316,521, 64316591–64316690, 64316920, 64316961, 64317641–64317682, and 64317769–64317870. Furthermore, we have also validated our gene prediction results using the Augustus version 3.3.3. The result obtained from the Augustus server https://bioinf.uni-greifswald.de/augustus/submission.php was similar to Gnomon prediction.The genetic organization and structure of the identified MADS box consisting of seven exon and six introns have been constructed using the Gene Display Server 2.0 (Fig. 6a-III). These findings provide valuable insights into the genetic makeup of these transcription factors, paving the way for deeper investigations into their functional roles in tomato growth and development.

### DNA motif analysis

Transcription factors represent the proteins that specifically bind with the cis- DNA elements to control the expression of other genes. Different hormones, such as auxins, gibberellins, cytokinins, abscisic acid, and ethylene, signal through specific transcription factors. These transcription factors recognize and bind to their corresponding DNA motifs in the promoter regions of target genes. DNA motifs associated with transcription factors allow for the integration of multiple hormonal signals. In many cases, a single transcription factor can be activated by multiple hormones.This enables the plant to respond dynamically to changing environmental conditions.The function of a novel DNA motif can be predicted by its comparision with database of recognized motifs and it can be predicted that if the DNA motif resembles a known transcription factor binding pattern it might be involved in controlling the gene expression under specific conditions. We have shown the potential DNA motifs associated with NAC domain containing protein. The full length gene prediction was done for NAC and other homologes. DNA motif search against the *Arabidopsis* JASPAR database using TOMTOM tool at the default settings. We reported three putative DNA motifs including CCGAACG GYTCACGGCCRAAYMGAGCDG (Motif1) (Fig. 6b-I), GCTTGAYGATTGGGTWTTRTGTCGAATMTACAAYAAGA (Motif 2) (Fig. 6b-II) and GGATWATGCACGARTAYCGMCTYGCYMAYGTKGAY (Motif 3) associated with the queried transcription factor/protein which resembled multiple transcription factor binding sites (Fig. 6b-II). These sites included homebox 53 (with a *p*-value of 6.39e-03), which is a member of HD-ZIP1 most closely related to HB53. AtHB53 is auxin-inducible and its induction is inhibited by cytokinin, especially in roots, indicating its potential involvement in root development. Another binding site was TCP family transcription factor (with a *p*-value of 3.70e-03), and NAC domain containing protein 62, a transcription factor that serves as a molecular link between cold signals and pathogen resistance responses.The third motif matched with AP2/ERFBP transcription factor (*p*-value-2.90e-04) and involved in encoding a member of the AINTEGUMENTA-like (AIL) subclass of the AP2/EREBP family of transcription factors and is essential for quiescent center (QC) specification and stem cell activity. In this way, motif analysis results identified DNA motifs resembling known transcription factor binding patterns for NAC transcriptional activators and their functional relevance and cross-talk with other transcriptional activators in a complex signaling network. These motifs suggest potential roles in diverse biological processes. This underscores the significance of motif analysis in understanding gene expression regulation.

### Co-expression analysis of DEGs

WCGNA based co-expression network was analyzed for only significant DEGs 1786 genes (*p*_cal_-value < 0.05) which constituted the gene dendogram with dynamic tree cut dividing the genes into differently colored modules. In our results, WCGNA analysis divided 1783 genes into a total 26 different colored modules. The maximum number of genes was reported in torquoise segment (179 genes) followed by purple (71 genes) and green-yellow (68 genes) and so on. The WCGNA analysis sorted the genes based on their functional annotation structured around three ontologies biological process, molecular function, and cellular component, KEGG pathway [91, 92] and all other enrichment databases. However, we identified, characterized and sorted the significant genes co-expressed in this network based on their FDR corrected *p*_adjusted_-values. Overall, in this co-expression network, 169 genes (*p*_adj_-value- 2.5e-38) were functionally classified or associated with metabolic pathways, 105 genes were found to be associated with biosynthesis of secondary metabolites (*p*_adj_-value 5.1e-25), 135 genes with small molecule metabolic process (*p*_adj_-value 1.8e-23), 320 genes co-expressed in metabolism of organo-nitrogen compound (*p*_adj_-value 2.1e-21), 135 genes involved in small molecule metabolic process (*p*_adj_-value 1.8e-23), 269 genes involved in biosynthetic processes (*p*_adj_-value 7.6e-15), 197 genes co-expressed in a cluster with function response to stimulus (*p*_adj_-value 5.7e-21), 174 genes co-expressed and involved in oxido-reductase activity (*p*_adj_-value 5.7e-21), 115 genes (*p*_adj_-value 4.2e-15) associated with stress response, 35 genes involved in carbon metabolism (*p*_adj_-value 1.8e-14). Interestingly, in the cellular component gene ontological term, we reported a maximum 392 genes (*p*_adj_-value 6.8e-50) located in cytoplasm followed by 110 genes (*p*_adj_-value 9.9e-19) located in plastid, and 109 genes (*p*_adj_-value 3.3e-19) along with others. Moreover, co-expression network constructed based on KEGG pathway [91, 92] revealed the similar results including 169 genes (*p*_adj_-value 2.1e-39), followed by 105 genes co-expressed simulataneously or interacting with other genes relevant to biosynthesis of the secondary metabolites (*p*_adj_-value 3.3e-26), 35 genes (*p*_adj_-value 2.1e-39) involved in carbon metabolism, 23 genes (*p*_adj_-value 3.3e-26), co-expressed and were relevant to amino acid biosynthesis. Overall we can conclude that the WCGNA based co-expression network revelead the enrichment of genes with maximum hit related to carbohydrate metabolism, secondary metabolite biosynthesis, and nitrogen metabolism during *Trichoderm*a interaction with tomato.

### Protein–protein interaction and network analysis

Protein–Protein interaction network was constructed for finding the hub and bottleneck genes with differential expression. We constructed the protein network for both upregulated (*p*_adj-_value < 0.05; FC > 1) and down-regulated (*p*_adj_-value < 0.05; FC < 1) DEGs. The topological properties and dynamics of this PPI network are highly modular, consisting of tightly interacting proteins that correspond to functional modules or protein complexes. Since the transcriptome data is highly dynamic representing differential expression of thousand of genes regulating multiple functions simultaneously and potentially associated with several functional categories. Finding the list of genes that are differentially expressed and co-expressed at a sime time could be usually inferred from protein–protein interactions. Moreover, finding the hub genes encoding hub proteins represent essential genes that are evolutionary conserved and affect the topological dynamics of the PPI network. Infact, topological dynamics of the PPI network depends on various parameter like selection of the genes foe which PPI network is required, confidence score at which the netwok had been analyzed, presence of specific gene/protein, presence or absence of other additional nodes from STRING database to predict the functional enrichment associated with a set of genes significantly. So, the main motto of analyzing the protein–protein interaction network in this work is to find the hub bottle-neck node, hub non-bottleneck node along with non-hub bottleneck node or genes or the genes that are differentially expressed or differentially co-expressed at a time to regulate the specific function of one or more than one specific pathways. These differentially expressed or co-expressed genes can be grouped separately into a specific cluster of the PPI network constructed with as significant functional enrichment. For PPI network analysis based on FC > 1 top 25 significantly expressed genes were selected. However, at a high confidence interval, non of them reported to have any mutual interactions suggesting that these selected genes were involved in regulating the distinct metabolic pathways or denoting different functional categories structured around three different ontologies where any node representing a specific functional category could have other possible nodes or genes regulating or similar pathway or co-expressed with other genes encoding protein participating in the same pathway or might have possibly involved in making interactions with other nodes for their shared function in different functional pathways. This become more clear when we add additional nodes or proteins in the PPI network from STRING database. The K mean clustering results re-construct the PPI network by dividing the entire network into one or more separate clusters depending on their functional relevance. K means clustering for the upregulated genes divided the PPI network into three separate clusters based upon the PPI partners and their associated function or pathway specific function enrichment including key interacting nodes regulating the amino acid metabolism pathway like Threonine synthase, (Solyc03g121910.1.1) Homoserine kinase (Solyc04g008760.1.1), and Alanine-glyoxylate transaminase / serine-glyoxylate transaminase / serine-pyruvate transaminase (Solyc12g099930.1.1) in the cluster 1 and all were involved in regulating the biosynthesis or degradation of various amino acids (red cluster) Similarly, cluster 2 involved all the interacting nodes playing a critical role in biosynthesis of branched achain amino acids with key partners like Acetolactate synthase (ALS) (Solyc03g044330.1.1), 3-isopropylmalate dehydratase (Solyc09g090900.2.1), Threonine dehydratase (Solyc09g008670.2.1) and others. However, the PPI network generated with significant number of genes/nodes represent all the possible functional KEGG pathways [91, 92] or gene-ontology annotations where the proteins might play an essential role or might have contributed partially to regulate the multiple pathways in a complex PPI network.

**Fig. 7:**
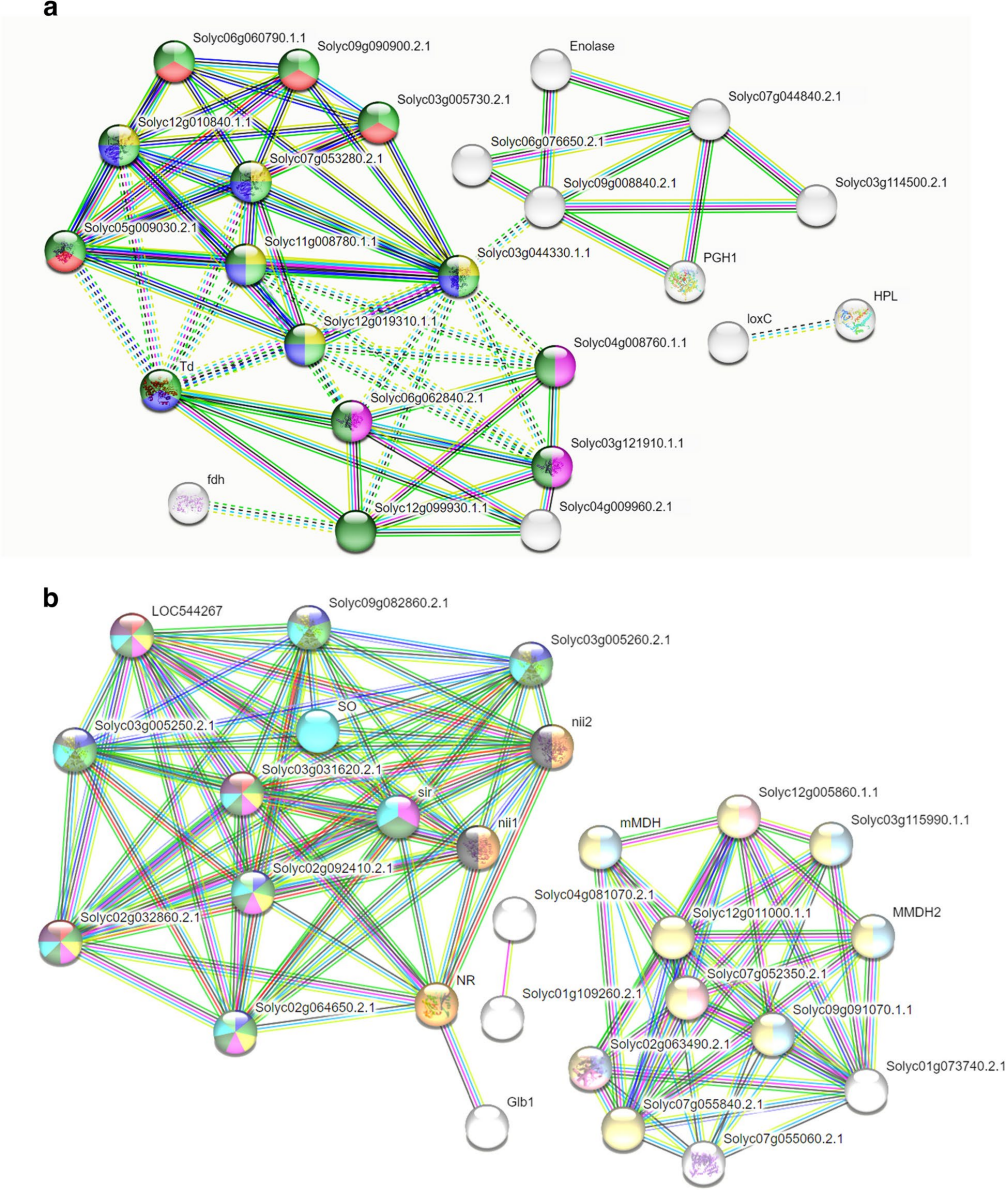
The STRING based protein–protein interaction network for both upregulated and the down-regulated genes. **a** PPI network for the topmost 25 genes with significant expression (*p*_adj_ < 0.05; *p*_cal_ < 0.05 and *p*_cal_ < 0.01and FC > 1). The colored ball indicated the genes/nodes along with edges. The differently colored ball indicated the shared contribution of each and every node with different proteins based on GO biological process involved. The k means clustering algorithm separated the entire PPI network into three different clusters and the separted clusters are shown with dotted line along the edges. **b** PPI network for the downregulated 25 genes with significant expression (*p*_adj_ < 0.05; *p*_cal_ < 0.05 and *p*_cal_ < 0.01, and FC < 1). The colored ball indicated the genes/nodes along with edges. The differently colored ball indicated the shared contribution of each and every node with different proteins based on GO biological process involved

**Fig. 8:**
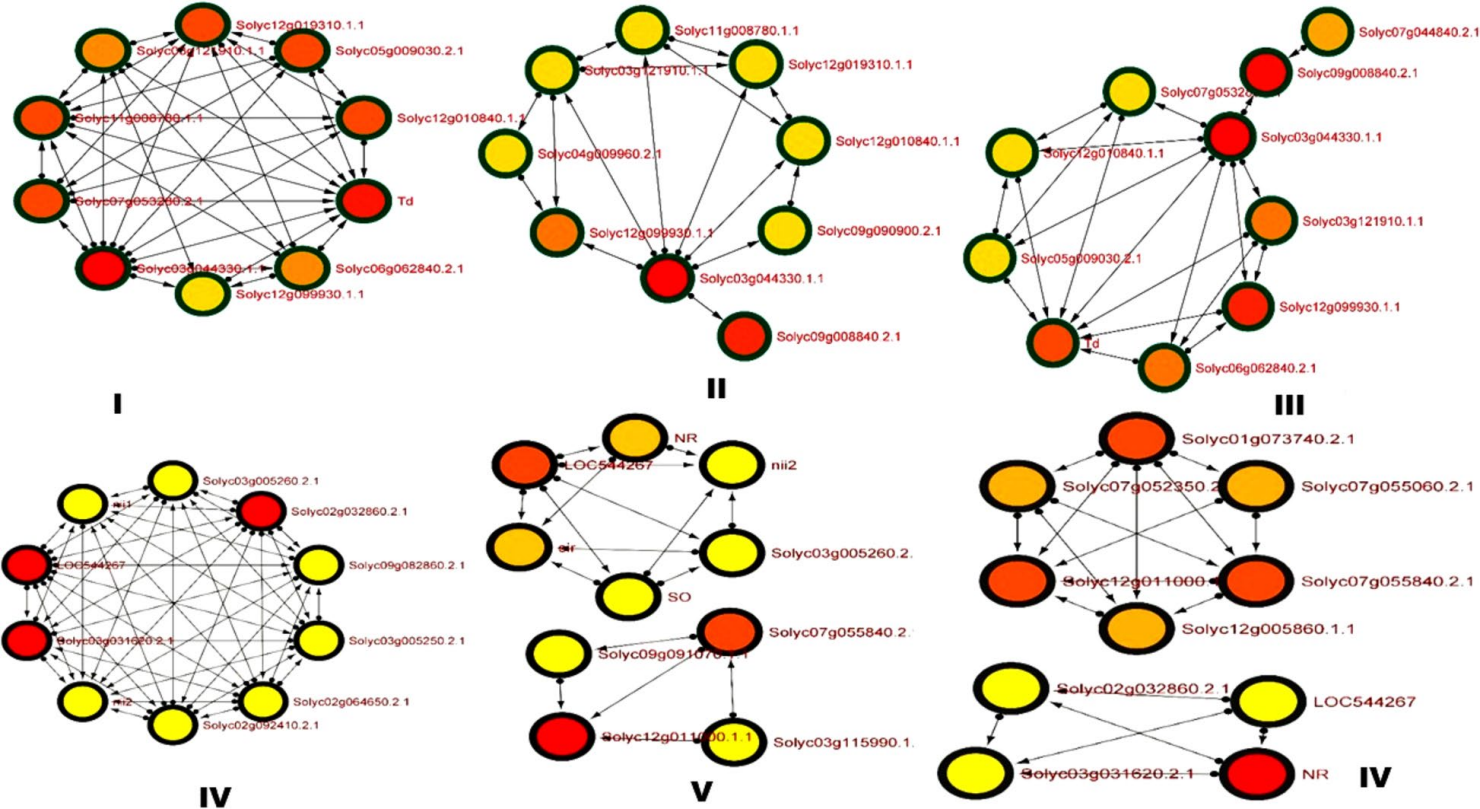
The Cytoscape based interaction network analyzing the ranking of the interacting partners based on the degree of interaction, betweenness centrality, and bottleneck approach to identify the hub and bottleneck genes. **I** PPI network for upregulated genes imported to Cytohubba plugin showing the topmost hits based on degree of interaction **II**. bottleneck approach **III**. betweenness centrality **IV**. PPI network for down-regulated genes imported to Cytohubba plugin showing the 10 topmost hits based on degree of interaction **V**. hits obtained through bottleneck approach. **VI**. hits obtained from betweenness centrality

In our results, we constructed the PPI network from topmost 25 significant and upregulated genes (*p*_adj_-value < 0.05) sorted based on FC > 1 (Fig. 7a). The PPI network from topmost 25 down-regulated genes (*p*_adj_-value < 0.05) and FC < 1 sorted based on STRING PPI network has been shown (Fig. 7b). The PPI network anlysis result with a functional enrichment value 1.11E-16 and average clustering coeffiecient 0.538 and calculated based on degree of interaction identified T_d_ (threonine dehydratase) as hub non-bottleneck node and was significantly expressed. Threonine deaminase/dehydratase (TD) functions as a housekeeping enzyme to convert threonine to α-ketobutyrate and ammonia as the committed step in the biosynthesis of isoleucine (Ile). Besides its housekeeping function in Ile biosynthesis, TD2 also plays a defensive role against insect herbivores and necrotrophic pathogens. Moreover, during plant-pathogen interactions, SA and JA signaling work antagonistically to each other TD2 regulates defense-related hormone crosstalk between SA and JA. Nevertheless, based on betweenness centrality and closenesss centrality and bottleneck approach from Cytohubba plugin revealed Solyc03g044330.1.1(acetolactate synthase) as hub-bottleneck node involved in biosynthesis of branched chain amino acids and was connected with non-hub bottleneck node Solyc09g008840.2.1(pyruvate kinase) that was expressed differentially (*p*_cal_-value < 0.05; FC 0.975). We have shown the STRING based PPI network for the upregulated genes in (Fig. 7a). The STRING based PPI network at a significant level of confidence score values as visualized in Cytoscape has been shown in Fig. S6. Furthermore, Cytoscape based network analyzed on degree of interaction (Fig. 8-I), betweenness centrality (Fig. 8-II), and the bottleneck approach have been shown (Fig. 8-III).

**Fig. 9:**
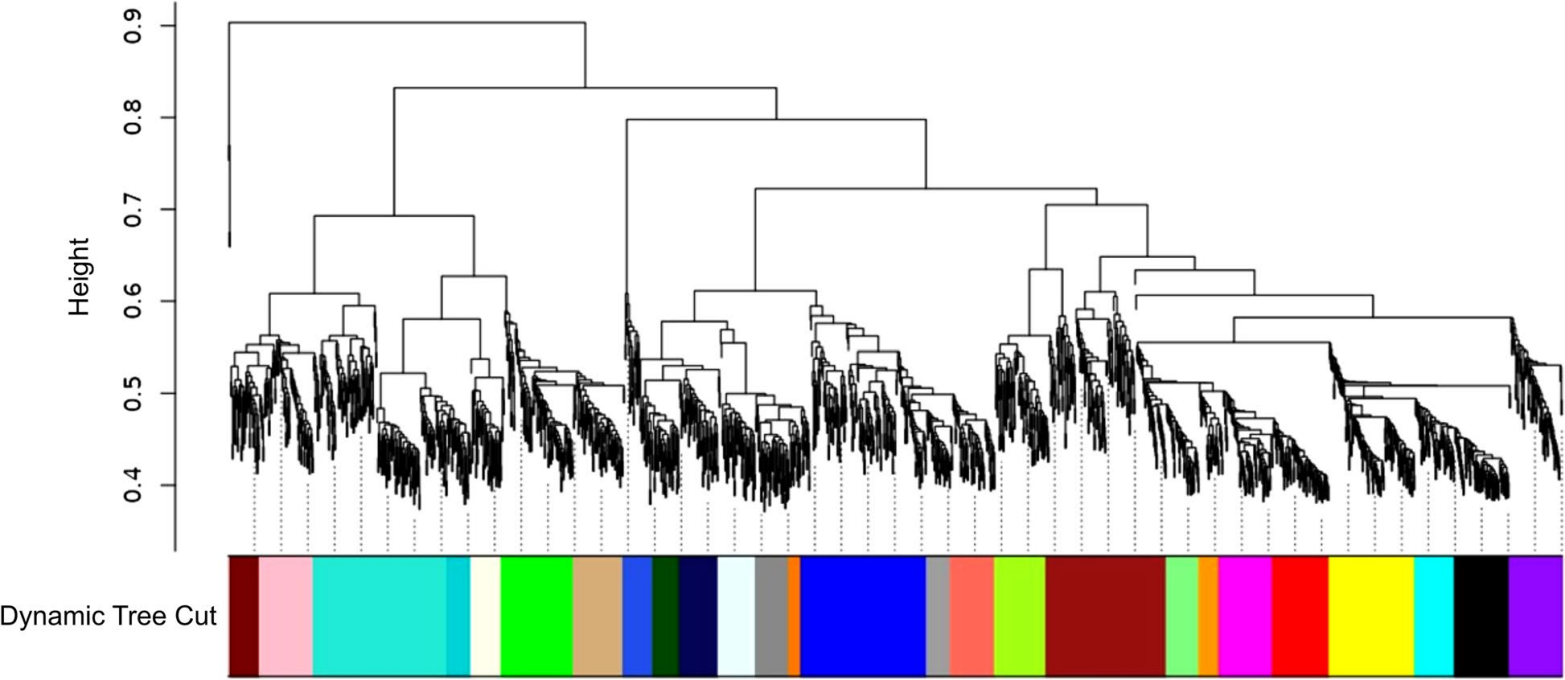
R-based WCGNA co-expression network showing the clustering of the 1786 significant DEGs (*p*_cal_-value < 0.05) into 26 modules with different colors and each color representing the list of participating genes of the specific and enriched GO terms along with FDR values

The PPI network for topmost downregulated 20 genes have been shown in (Fig. 8-IV) The network analyzer results for topmost downregulated genes showed that based on degree of interactions the topmost hub nodes reported were ferredoxin–nitrite reductase (chloroplastic) nii1 (Solyc01g108630.2.1), Sulfite reductase (ferredoxin) (Solyc11g065620.1.1), Citrate synthase (Solyc12g011000.1.1) (FC > 1; *p*cal-value < 0.05). However, based on betweenness centrality and bottleneck approach, nitrate reductase (NADPH) was found as hub bottleneck node. The hub nodes based on degree of interaction and identified through Cytoscape pluggin were adenylyl-sulfate reductase (glutathione) (LOC544267), thioredoxin domain-containing protein (Solyc02g032860.2.1) (FC > 1), Ferredoxin–nitrite reductase (nii2) (FC < 1; *p*_cal_-value < 0.05), sulfate adenylyltransferase (Solyc03g005260.2.1) and Solyc09g082860.2.1), adenylyl-sulfate kinase (Solyc02g092410.2.1), nii1, and Solyc02g064650.2.1 (Fig. 8-IV). However, based on betweenness centrality NR (Solyc11g013810.1.1) (FC < 1; *p*_cal_-value < 0.05) was reported to be hub bottleneck node (Fig. 8-V). In contrast, bottleneck method for top 10 hits reported Citrate synthase (Solyc12g011000.1.1), and adenylyl-sulfate reductase (glutathione) (LOC544267) (FC < 1; *p*_cal_-value < 0.05) as bottleneck node followed by mitochondrial Malate dehydrogenase (Solyc07g055840.2.1) (FC > 1; *p*_cal_-value < 0.05) as non-hub bottleneck node (Fig. 8-VI). We found that among the down-regulated genes the hits obtained for genes involved in metabolism of sulphur, purine metabolism, selenocompound metabolism, Monobactam biosynthesis, Biosynthesis of secondary metabolites and nitrogen metabolism were more prevalent in addition to the hits involved in common metabolic pathways. K means clustering for the PPI network into functional enrichment for 2 clusters including genes involved in Nitrogen metabolism (sir, nii1, nii2, and NR) and Sulphur metabolism like Solyc02g094120.2.1, Solyc03g031620.2, Solyc02g080640.2.1, Solyc03g031620.2.1, Solyc02g064650.2.1, and Solyc03g005250.2.1. Overall, PPI interaction network analysis revealed the enrichment of proteins involved in biosynthetic mechanisms of metabolites, oxido-reductive changes and assimilation and metabolism of nitrogenous compound in tomato when supplemented with *Trichoderma* spp. Interestingly, our results based on protein–protein interactive association network highlighted the relevance of target proteins with other interacting proteins along with their functional aspects (interacting partner). We have shown the WCGNA based co-expression network for the 1786 significant DEGs (*p*_cal_-value < 0.05) (Fig. 9).

### Gene ontology and functional annotation

Gene ontology terms are descriptions of the gene products that are structured around three ontological terms including molecular function, cellular component, and biological processes involved. In our results, GO enrichment analysis, in terms of biological processes involved the topmost five significant terms based on FDR enrichment were response to stress (GO:0006950), response to stimulus (GO:0050896), defense response (GO:0006952), response to biotic stimulus (GO:0009607), cellular process (GO:0009987), metabolic process (GO:0008152). Besides, regulation of molecular function (GO:0065009), organic substances metabolic process (GO:0071704), defense response to fungus (GO:0050832), negative regulation of peptidase activity (GO:0010466), regulation of proteolysis (GO:0030162), negative regulation of peptidase activity (GO:0010466). However, among the biological processes GO term, we encountered maximum number of genes (18) and pathway genes (301) belonging to cellular process (GO:0009987) with significant enrichment FDR (3.5E-09) followed by metabolic process (FDR, 4.7E-09) capturing 17 genes and 271 pathway genes. The enriched GO terms associated with significant DEGs and structured around ontological terms like biological process, molecular function and KEGG pathway [98, 99] has been represented through tree-map diagrame of ReviGO tool (Fig. S7 and S8).

In GO molecular function, the topmost enriched terms found were catalytic activity (GO:0003824), binding (GO:0005488), hydrolase activity (hydrolyzing O-glycosyl compounds; GO:0004553), molecular function regulator activity (GO:0098772), endopeptidase inhibitor activity (GO:0004866), peptidase regulator activity (GO:0061134), endopeptidase regulator activity (GO:0061135), hydrolase activity (GO:0016787), enzyme regulator activity (GO:0030234), enzyme inhibitor activity (GO:0004857). Furthermore, in molecular function GO term maximum number of genes and pathways with significant FDR found were catalytic activity with 18 genes and 229 pathway genes (FDR, 6.6E-11) followed by binding 16 genes and 249 pathways (FDR, 1.3E-08).

In cellular component, topmost hits retrieved were cellular anatomical entity (GO:0110165), extracellular region (GO:0005576), intracellular anatomical structure (GO:0005622), membrane-bounded organelle (GO:0043227), intracellular membrane-bounded organelle (GO:0043231), cytoplasm (GO:0005737), organelle (GO:0043226). Likwise, the biological process and molecular function GO term, in cellular component, maximum genes with significant enrichment (FDR, 4.6E-19) reported were 29 genes and 346 pathways genes and belonging to cellular anatomical entity (GO:0110165) followed by genes intracellular (17 genes and 290 pathway genes with significant FDR 5.4E-09), organelle (7 and 244, FDR, 1.0E-07), cytoplasm (7 and 245, FDR, 1.0E-07).

Nevertheless, STRING based PPI network analysis for topmost upregulated and downregulated genes identified and characterized the functional enrichment network based on significant FDR, count in network, and strength of the network that was constructed based on both shell of protein interacting partners and their shared functions structured around specific ontological terminologies. The different colored nodes represent the unique functional aspect shared by each interactive partner with others. For example, we found the heme binding function (GO:0020037) was shared by Solyc09g061230.2.1 along with Solyc06g007930.2.1, Solyc06g083440.2.1, nitrite reductase, Solyc01g087550.2.1, Solyc09g061230.2.1, non-symbiotic hemoglobin (Glb1) and two other non-hub and non-bottleneck nodes including Solyc08g068070.2.1 and Solyc08g068090.2.1. Likewise, functional annotation related to electron transfer reaction was shared by the Solyc09g061230.2.1, Solyc06g007930.2.1, Solyc06g083440.2.1. The function oxygen binding function was shared by Solyc08g068090.2.1, Solyc08g068070.2.1, Glb1. Functional annotation related to oxidoreductase activity was contributed by Solyc09g061230.2.1, Solyc00g033880.1.1, Solyc10g081440.1.1, Solyc06g007930.2.1, Solyc06g083440.2.1, Nitrite reductase, Solyc05g018520.2.1. Moreover, oxygen transport function is shared by Glb1, Solyc08g068070.2.1, Solyc08g068090.2.1. Based on KEGG pathway analysis [91, 92], maximum interactive association of protein networks were involved in biosynthesis of amino acids (sly01230) including valine, leucine, and isoleucine (sly00290), followed by biosynthesis of secondary metabolites (sly01110), C_5_ dibasic acid metabolism (sly00660), metabolism of amino acids, 2-oxo-carboxylic acid metabolism (sly01210), metabolic pathways (sly01100). The functional enrichment based on STRING based PPI network associated with the topmost 25 genes significantly expressed and upregulated DEGs structured around gene ontological terms like biological process, molecular function, and KEGG pathways [91, 92] has been shown in Table S2. The functional enrichment based on STRING based PPI network associated with the topmost 25 genes significantly expressed and downregulated DEGs structured around gene ontological terms like biological process, molecular function, cellular component, and KEGG pathways [91, 92] has been shown in Table S3. Overall, based on this protein–protein interactive association network we can predict that during *Trichoderma*-tomato interaction the fungal partner reprogramme the transcriptional machinery of host towards the biosynthesis of amino acid, carbohydrates, vitamins, secondary metabolites, and oxidative metabolism.

## Discussion

The beneficial plant-fungal interactions results into several advantages to the host plants including disease control, stimulation of plant growth, greater yield, improved nutrient bioavailability and uptake, and improvement of crop quality [127–129]. *Trichoderma* (Ascomycota, Hypocreales, Hypocreaceae) are widely distributed and frequently found in soil, as plant symbionts, saprotrophs, and mycoparasites [5]. A few species have been utilized to reduce unfavorable growing conditions and control a variety of plant diseases. *Trichoderma* interact favorably with plant roots, activating the plant immune system and promoting systemic defense against pathogen invasion. *Trichoderma* supplementation has been shown to have positive impacts on plants in terms of growth promotion and defense induction against biotic and abiotic challenges in addition to its biocontrol action [38, 42, 130]. Consequently, *Trichoderma* spp. treated plants may be bigger, healthier, and produce more than untreated plants [19].

Several studies targeting the transcriptomic or proteomic approaches for unravelling the *Trichoderma*-plant interactions or *Trichoderma*-root colonization have focused the transcriptional profiling, physiological or biochemical changes occurring mainly during the early phase of interactions [11, 24, 28, 51]. In one report, a high-density oligonucleotide microarray was used to evaluate the effect of *T. hamatum* 382 on expression of 15925 genes in leaves and the study reported that *T. hamatum* 382 supplementation resulted into differential expression of 45 genes in all the replicates out of which 41 genes were clustered separately into seven functional categories including metabolism of carbohydrates, protein metabolism, DNA and RNA metabolism, defense response, and stress tolerance [28]. In fact, *Trichoderma* supplementation or interaction with host tissue resulted into transcriptional re-programming of host genome leading to genome-wide expression of thousand of genes. However, all the genes that are expressed across the host genome are not statistically significant and/or the sorting the statistically significant genes based on *p*-value may give false positive results [131, 132]. While analyzing the microarray data finding significant genes that are differentially expressed (DEGs) across the two experimental conditions is an important step. These DEGs infact function as a molecular driving force or molecular biomarkers for different phenotypes and many methods have been used to sort the DEGs including combining *p*-value, fold change, and other statistical methods. However, a pre-determined cut-off score values is mandatory for implication of such methods [64]. If there is a statistically significant difference between the read counts of two experimental conditions or a change in the expression levels of a gene, the gene is declared as differentially expressed [133]. In hypothesis testing, the *p*-value is a measure of the strength of evidence against a null hypothesis. A common approach is to compare the *p*-value to a threshold (usually 0.01 or 0.05) to determine if the evidence is strong enough to reject the null hypothesis. A *p*-value below the threshold suggests that the association is statistically significant [133–135]. In fact, the *p*-value is a measure of the strength of evidence against the null hypothesis that there is no difference in gene expression between the two groups. Furthermore, identification of DEGs in a microarray data is a challenging task as selection and ranking of the DEGs based on *p*-value calculation and/or through fold change determination have their own pros and cons. The q value, like the *p* value, assigns a unique measure of significance to each feature. The *p* value is a measure of significance in terms of the false positive rate, whereas the q value is a measure in terms of the FDR. The false positive rate and FDR are sometimes confused, although the distinction is significant. Given a rule for calling features significant, the false positive rate is the proportion of times that actually null features are considered significant [133]. The FDR is the rate at which significant features are genuinely null. A false positive rate of 5%, for example, means that 5% of the actually null features in the study will be classified as significant. In contrast, a FDR of 5% indicates that, on average, 5% of the features considered significant are actually null [133–135]. In one report, Zhao et al. [64] reported the comparision of two different methods (*p*-value-based and FC-based) to sort DEGs. The study reported that genes with high *p*-values may have significant FCs while some top-list genes with low *p*-values may not have large FCs when using *p*-value to rank genes. On the other hand, top-list genes with large FCs may have high *p*-values when using FC to rank genes, and genes with tiny FCs may have low *p*-values. Generally, it has been assumed that probability value with cut-off (*p*_cal_-value < 0.05) is considered as standard for finding the differentially expressed and significant genes [69]. Nevertheless, sorting of the significant genes at the cut-off (*p*_calculated_-value < 0.05) is utterly arbitrary and just serves as a convention as a specific measure associated with each gene would be worthwhile [134]. The choice of threshold can affect the number of genes identified as differentially expressed and the false discovery rate (the proportion of false positives among the genes identified as differentially expressed) and therefore, could not be suitable for all variables and research contexts, particularly, for disease association studies, where the lower cut-off of 0.01 (*p*_cal_-value < 0.01 is generally recommended [73, 135]. In one report, Andarde [73] reported that setting a threshold limit for determination of a statistically significant value is useful, its limitation should be considered and selection of a threshold cut-off score below the widely used 0.05 and measuring the false positive rate could be a good approach. Moreover, adjusted *p*-values for a multiple testing method that tightly regulates the Type I error rate and takes into account the dependence structure between the gene expression levels are used to identify DEGs [131]. No specific parametric form is assumed for the distribution of the test statistics and a permutation procedure is used to estimate adjusted *p*-values. In one report, its has been suggested that the number of DEGs that are significant and are differentially expressed is highly dependent on sample size and variability. On average, even with low-quality or spatially different samples, the amount of DEGs showed less variance the more samples that were used. With various FDR-adjusted *p* value cut-offs (*p*_adj_-value < 0.05, *p*_adj_-value < 0.01, *p*_adj_-value < 0.1), the outcome was not significantly changed [136]. Overall, it has been suggested that when analysing microarray data, DEGs can be chosen by combining *p*-value and fold-change (FC) [137]. We can conclude that the research question, the quality of the data, and the likelihood of false positives and false negatives should all be carefully taken into account when deciding on the threshold for identifying differential expression.

In our results, out of 10209 probe sets deposited on microarray, only 329 genes were reported to be differentially expressed at a *p*_adj_ < 0.05 under the *T. harzianum* treatment condition and 1786 genes differentially expressed across the replicated treatments with (*p*_cal_-value < 0.05). However, at a much lower stringent cut-off score values (*p*_cal_ < 0.001) we found only 56 genes that were differentially expressed along with 26 upregulated (FC > 1) and 30 downregulated genes (FC < 1) and the genes retrieved were also found in FDR corrected *p*_adj_-value < 0.05 group. Our results is further supported by results and transcriptiome data *T. afroharzianum* interaction with tomato roots post colonization resulted into the differential expression of 984 DEGs playing critical role in metabolic pathways, biosynthesis of phenylpropanoids (secondary metabolism), glutathione metabolism (oxidative stress), phytohormone homeostasis [138]. In our results, based on gene ontology structured around biological process term the significant DEGs (*p*_cal_-value < 0.001) were enriched around several functional categories like positive regulation of oxidative phosphorylation, dimethylallyl diphosphate biosynthesis (building block for the biosynthesis of a wide variety of isoprenoid compounds, including sterols, carotenoids, and terpenes), isopentenyl diphosphate biosynthesis, (isoprenoid biosynthesis), sterol biosynthesis, pyruvate metabolism, organic hydroxy compound biosynthesis, isoprenoid biosynthesis, lipid biosynthetic and metabolism, cellular biosynthesis. While in the molecular function term we found hits for functional activities phosphoglycerate kinase (carbohydrate metabolism), diphosphomevalonate decarboxylase (DPMVD) involved in isoprenoid biosynthesis, and structural constituent of ribosomes. Furthermore, based on KEGG pathway, the major pathways reported were associated with functional categories like biosynthesis of terpenoids, sterol, carbohydrate metabolism, carbon fixation, biosynthesis of amino acids, secondary metabolites, and metabolic pathways. However, we also reported the differential expression of host-defense related PR proteins including PR 2, PR 3, PR 5 and PR 4 in association with growth, development and defense-related transcription factors like MYB, NAC, and MADS box in *Trichoderma* treated samples. Microbial priming with *Trichoderma* results into activation of plant defense through phytohormonal cross-talk and involves SA, JA, and ET [139]. Further, differential expression of various transcription factors like MYB, bHLH, NAC, WRKY, and the MYCs play an essential role in priming response as these transcription factors function as regulatory switch of the transcriptional network for stress induced systemic defense [140]. Moreover, significant expression of plant defense-related PR genes is further confirmed through differential expression of hormone-responsive marker genes. In one study, accumulation of SA and expression of SA-responsive marker genes like PATHOGENESIS RELATED-1a (PR-1a) have been reported in *Arabidopsis*-*T. virens* interaction [141]. Recently, induction of 16 different types of PR proteins including PR1, PR5, PRP2, PR10, PRSTH-2/PRSTH-2 like, chitinases, and glucan 1, 3-β-glucosidases have been reported to be significantly upregulated in *Botrytis cinerea* derived cell wall degrading enzymes (CWDEs) [142]. The results from our study is well supported by work of Coppolla et al. [143] who reported the the transcriptomic and metabolic reprogramming of tomato defense against aphids as *T. harzianum*-aphid-tomato tripartite interaction resulted into differential expression of both early and late genes acting directly or indirectly in host defense against aphids like Chitinases, GST kinases, Polyphenol oxidases, Peroxidases along with overexpression of tomato defense-realted. transcription factors like WRKY, NAC, bZIP, AP2/ERF that might have played a key role in priming response of the host by fungus against aphids. Moreover, defense genes that work indirectly were generally involved in regulating the secondary metabolism like Sesquiterpene synthase and Geranylgeranyl phosphate synthase. Furthermore, metabolic re-programming results revealed the biosynthesis and accumulation of isoprenoids in *Trichoderma* treated plants which further support our results. Likewise, Yuan et al. [34] reported that *T. longibranchiatum* H9 induced systemic resistance in cucumber involves the key role of salicylic acid (SA), jasmonic acid (JA) and ethylene (ET). Transcriptomic characterization and GO analysis to classify the function of DEGs both upregulated and downregulated during *T. longibranchiatum* H9 interaction with cucumber revealed enrichment of pathways related to biosynthesis of Phenylpropanoids, Flavonoids, Phytohormones, Phenylproapanoid metabolism (associated with biosynthesis of (SA), cysteine and methionine metabolism (ET biosynthesis), signal transduction, and pathways related to defense and stress response. Further, based on KEGG enrichment pathways [98, 99] that showed the topmost hits during *T. longibranchiatum* H9-cucumber interaction were related to biosynthesis of secondary metabolites, biosynthesis of branched chain amino acids, metabolism of alpha linoleic acid, fatty acid metabolism, and metabolic pathways which further support our study.

## Conclusion

The present study intricately explored the interplay between *Trichoderma* and the tomato genome elucidating the multifaceted interplay attributed to the impact of microbial priming. Depositing RNA-seq datasets in the Gene Expression Omnibus (GEO) [144] and Sequence Read Archive (SRA) [145] repositories ensures the reproducibility and reuse of published findings. These data can be reanalyzed to yield new scientific insights [146]. The transcriptomic characterization/microarray profiling based on statistical methods including adjusted *p*-values (FDR corrected) and raw *p*-values (p-calculated) represents a robust approach to find a reliable set of differentially expressed genes (DEGs) regulating the metabolic alterations, biochemical changes, and plant growth promotion attributes required during late colonization events, and therefore, diverting the cellular energy for plant growth and development at the cost of host defense. Moreover, identified and characterized common set of genes signify their biological relevance, making them prime targets for deeper molecular and functional analyses. In fact, microbial priming with *Trichoderma* led to a rewiring of the transcriptional activities of the host genome that resulted into differential expression of a specific group of genes and proteins regulating the transcriptional responses. Interstingly, the significant genes that were differentially expressed, co-expressed, and/or involved in a complex protein–protein interaction network highlighted their relevance in regulating the crucial pathways such as carbohydrate metabolism, secondary metabolite biosynthesis, and nitrogen metabolism. The study also highlights the complex molecular mechanisms orchestrating *Trichoderma*-induced modulation of tomato gene expression, providing insights into the impact on metabolic pathways and biochemical changes during late colonization. The utilization of statistical methods enhanced our understanding of regulatory networks governing host responses to beneficial microbial interactions, contributing to the advancement of knowledge in plant–microbe interactions with potential implications for sustainable agriculture.

### Supplementary Information

**Additional file 1: Figure S1.** Multidimensional scaling analysis of the gene expression data from both un-inoculated control (C) and treatment (T) to map the high dimensional data into two dimensions while keeping the relative distances between the observations constant. **Figure S2.** t-SNE plot displaying the points from a higher dimension to a lower dimension trying to preserve the neighborhood of that point. t-SNE plot t-SNE is also a un-supervised non-linear dimensionality reduction and data visualization. **Figure S3.** Pathway analysis of PCA rotation based on functional enrichement structured around biological process GO term. **Figure S4.** Pathway analysis of PCA rotation based on functional enrichement structured around molecular function GO term. **Figure S5.** Pathway analysis of PCA rotation based on functional enrichement based on Kyoto Encyclopedia of Genes and Genome(KEGG) pathway. The functional annotation and gene-specific pathway for the significant and associated hits were retrieved through ShinyGO based KEGG tool [91, 92]. **Figure S6.** Cytohubba constructed PPI network for upregulated genes showing the interactive associative network. **Figure S7.** Tree map based on Revi GO analysis showing the functional annotation of the enriched GO IDs associated with significant DEGs structured around gene ontological term biological process involved. **Figure S8.** Tree map based on Revi GO analysis showing the functional annotation of the enriched GO IDs associated with significant DEGs structured around gene ontological term molecular function. **Table S1.** Table showing the different values of multiple principle component analysis (PCAs), multidimensional scaling and t-SNE analysis for both un-incoculated control samples (C1, C2, and C3) and inoculated treatments(T1,T2, and T3). **Table S2.** Functional enrichment and annotation of the top 25 significant DEG (*p *cal-value <0.05; *p*cal-value <0.01, and *p *adj- value <0.05) and upregulated (FC >1) structured around the three ontological terms including biological process, molecular function, cellular component, and KEGG pathways. The PPI network was constructed based on high confidence interval with 10 additional nodes from database with significant enrichment value PPI enrichment (*p*-value < 1.0e-16). **Table S3.** Functional enrichment and annotation of the top 25 significant DEG (*p*cal-value <0.05; *p*cal-value <0.01, and *p*adj-value <0.05) and down-regulated (FC <1) structured around the three ontological terms including biological process, molecular function, cellular component, and KEGG pathways. The PPI network was constructed based on high confidence interval with 10 additional nodes from database with significant enrichment value PPI enrichment (*p*-value < 1.0e-1).

## Data Availability

All the data used in this manuscript are available online and can be checked on Nation Centre for Biotechnology Information (NCBI) Gene Expression Omnibus (GEO) platform with Bioproject ID GSE76332 (https://www.ncbi.nlm.nih.gov/geo/query/acc.cgi?acc=GSE76332).

## Abbreviations

DEGs: Differentially expressed genes
*P*_cal_-value: Calculated probability value
*P*_adj_-value: Adjusted probability value
FDR: False discovery rate
SA: Salicylic acid
PRRs: Pattern recognition receptors
SAR: Systemic acquired resistance
WCGNA: Weighted co-expression network
GO: Gene ontology
ESTs: Expressed sequence tags
PPI: Protein-protein interaction network
