# Supplementary material for: Transcriptomic characterization of Trichoderma harzianum T34 primed tomato plants: assessment of biocontrol agent induced host specific gene expression and plant growth promotion
Source: BMC Plant Biol. 2023 Nov 8;23:552. doi: 10.1186/s12870-023-04502-6 (PMC10631224; doi:10.1186/s12870-023-04502-6)
Supplement: Supplementary file 1 — Additional file 1: Figure S1. Multidimensional scaling analysis of the gene expression data from both un-inoculated control (C) and treatment (T) to map the high dimensional data into two dimensions while keeping the relative distances between the observations constant. Figure S2. t-SNE plot displaying the points from a higher dimension to a lower dimension trying to preserve the neighborhood of that point. t-SNE plot t-SNE is also a un-supervised non-linear dimensionality reduction and data visualization. Figure S3. Pathway analysis of PCA rotation based on functional enrichement structured around biological process GO term. Figure S4. Pathway analysis of PCA rotation based on functional enrichement structured around molecular function GO term. Figure S5. Pathway analysis of PCA rotation based on functional enrichement based on Kyoto Encyclopedia of Genes and Genome(KEGG) pathway. The functional annotation and gene-specific pathway for the significant and associated hits were retrieved through ShinyGO based KEGG tool [91, 92]. Figure S6. Cytohubba constructed PPI network for upregulated genes showing the interactive associative network. Figure S7. Tree map based on Revi GO analysis showing the functional annotation of the enriched GO IDs associated with significant DEGs structured around gene ontological term biological process involved. Figure S8. Tree map based on Revi GO analysis showing the functional annotation of the enriched GO IDs associated with significant DEGs structured around gene ontological term molecular function. Table S1. Table showing the different values of multiple principle component analysis (PCAs), multidimensional scaling and t-SNE analysis for both un-incoculated control samples (C1, C2, and C3) and inoculated treatments(T1,T2, and T3). Table S2. Functional enrichment and annotation of the top 25 significant DEG (p cal-value <0.05; pcal-value <0.01, and p adj- value <0.05) and upregulated (FC >1) structured around the three ontological ter [file 12870_2023_4502_MOESM1_ESM.docx]

**Supplementary Files**

**Figure S1** Multidimensional scaling analysis of the gene expression data from both un-inoculated control(C) and treatment (T) to map the high dimensional data into two dimensions while keeping the relative distances between the observations constant**.**

**Figure S2** t-SNE plot displaying the points from a higher dimension to a lower dimension trying to preserve the neighborhood of that point. t-SNE plot t-SNE is also a unsupervised non-linear dimensionality reduction and data visualization.

**Figure S3** Pathway analysis of PCA rotation based on functional enrichement structured around biological process GO term.

**Figure S4** Pathway analysis of PCA rotation based on functional enrichement structured around molecular function GO term.

**Figure S5** Pathway analysis of PCA rotation based on functional enrichement based on Kyoto Encyclopedia of Genes and Genome(KEGG) pathway. The functional annotation and gene-specific pathway for the significant and associated hits were retrieved through ShinyGO based KEGG tool [90],[91].

**Figure S6** Cytohubba constructed PPI network for upregulated genes showing the interactive associative network.

**Figure S7.** Tree map based on Revi GO analysis showing the functional annotation of the enriched GO IDs associated with significant DEGs structured around gene ontological term biological process involved.

**Figure S8.** Tree map based on Revi GO analysis showing the functional annotation of the enriched GO IDs associated with significant DEGs structured around gene ontological term molecular function.

**Table S1** Table showing the different values of multiple principle component analysis (PCAs), multidimensional scaling and t-SNE analysis for both un-incoculated control samples (C1, C2, and C3) and inoculated treatments(T1,T2, and T3).

**Table S2** Functional enrichment and annotation of the top 25 significant DEG (p_cal_-value <0.05; p_cal_-value <0.01, and p_adj_- value <0.05) and upregulated (FC >1) structured around the three ontological terms including biological process, molecular function, cellular component, and KEGG pathways. The PPI network was constructed based on high confidence interval with 10 additional nodes from database with significant enrichment value PPI enrichment (p-value < 1.0e-16).

**Table S3** Functional enrichment and annotation of the top 25 significant DEG (p_cal_-value <0.05; p_cal_-value <0.01, and p_adj_-value <0.05) and down-regulated (FC <1) structured around the three ontological terms including biological process, molecular function, cellular component, and KEGG pathways. The PPI network was constructed based on high confidence interval with 10 additional nodes from database with significant enrichment value PPI enrichment (p-value < 1.0e-1)

**Supplementary Figures**

**Figure S1**


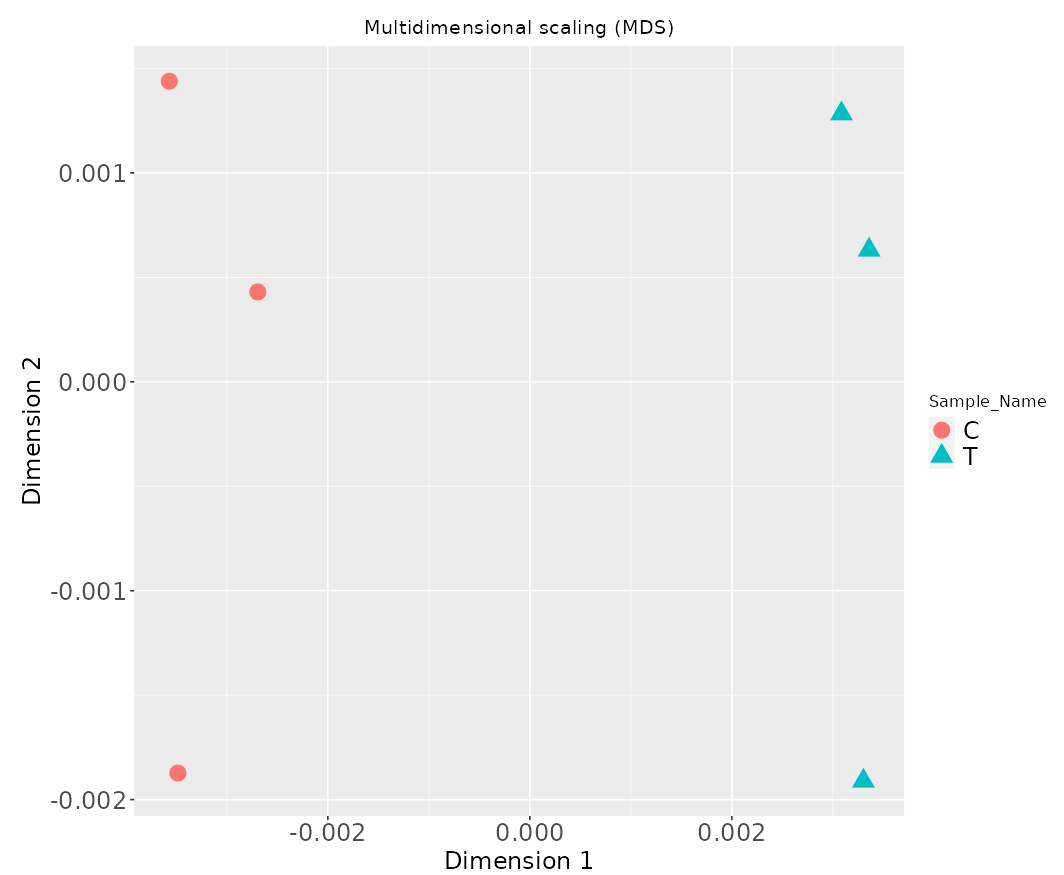


**Figure S2**


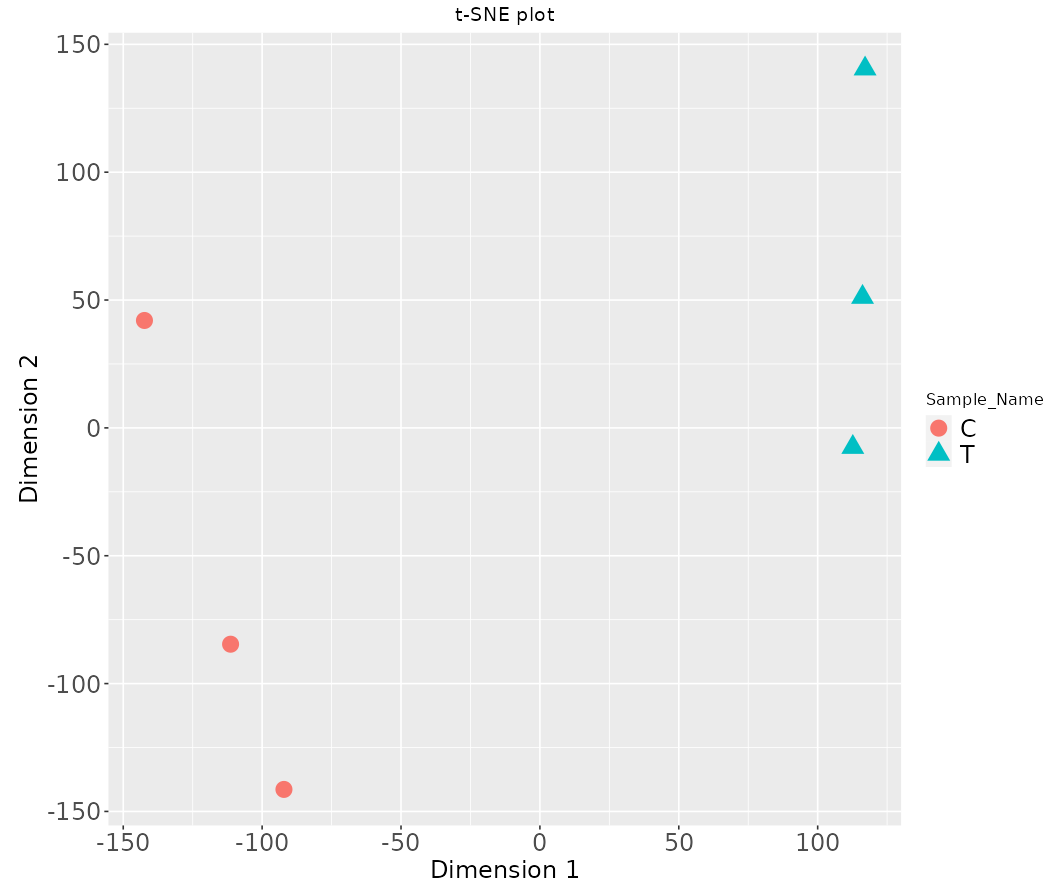


**Figure S3**


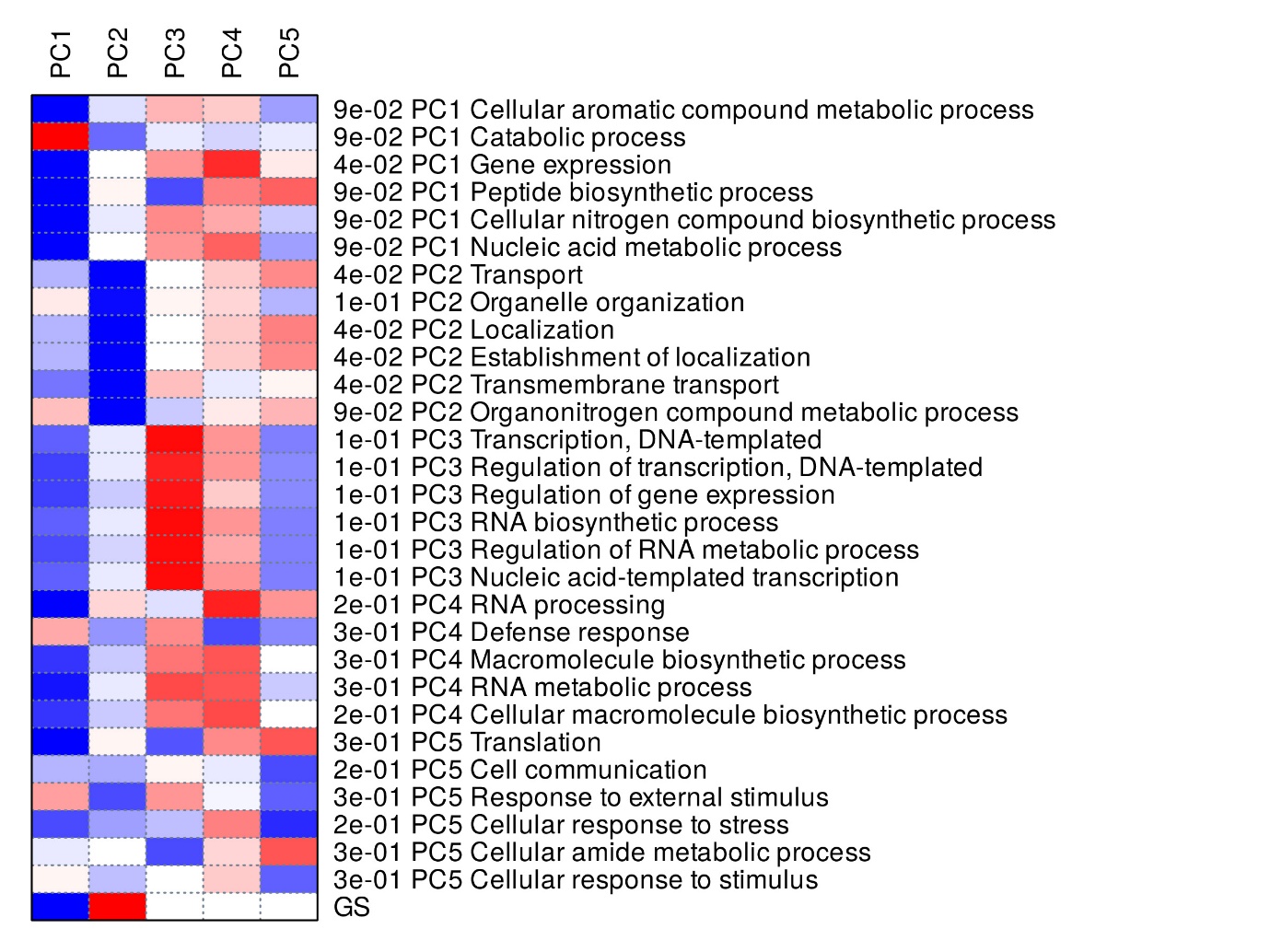


**Figure S4**


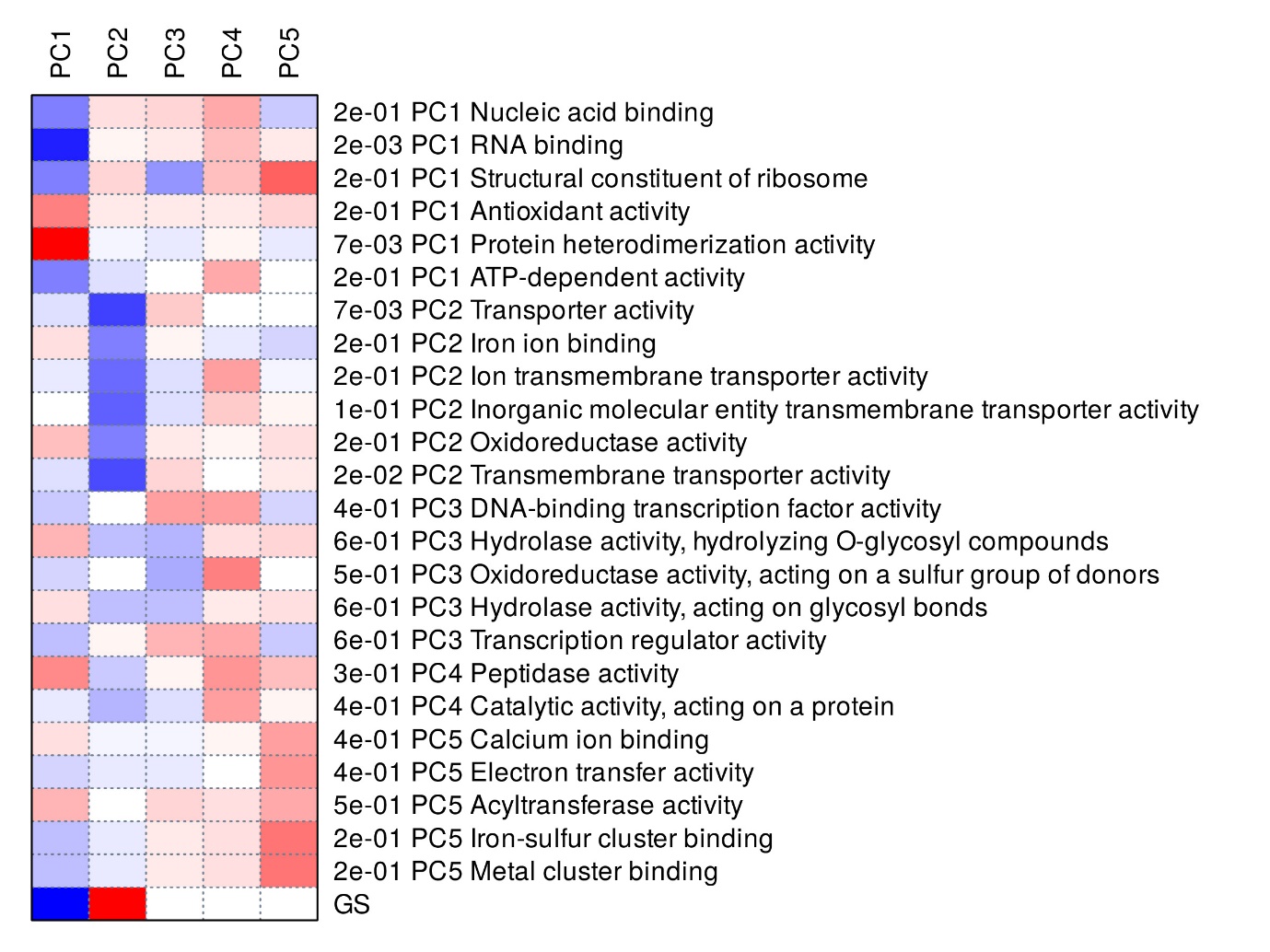


**Figure S5**


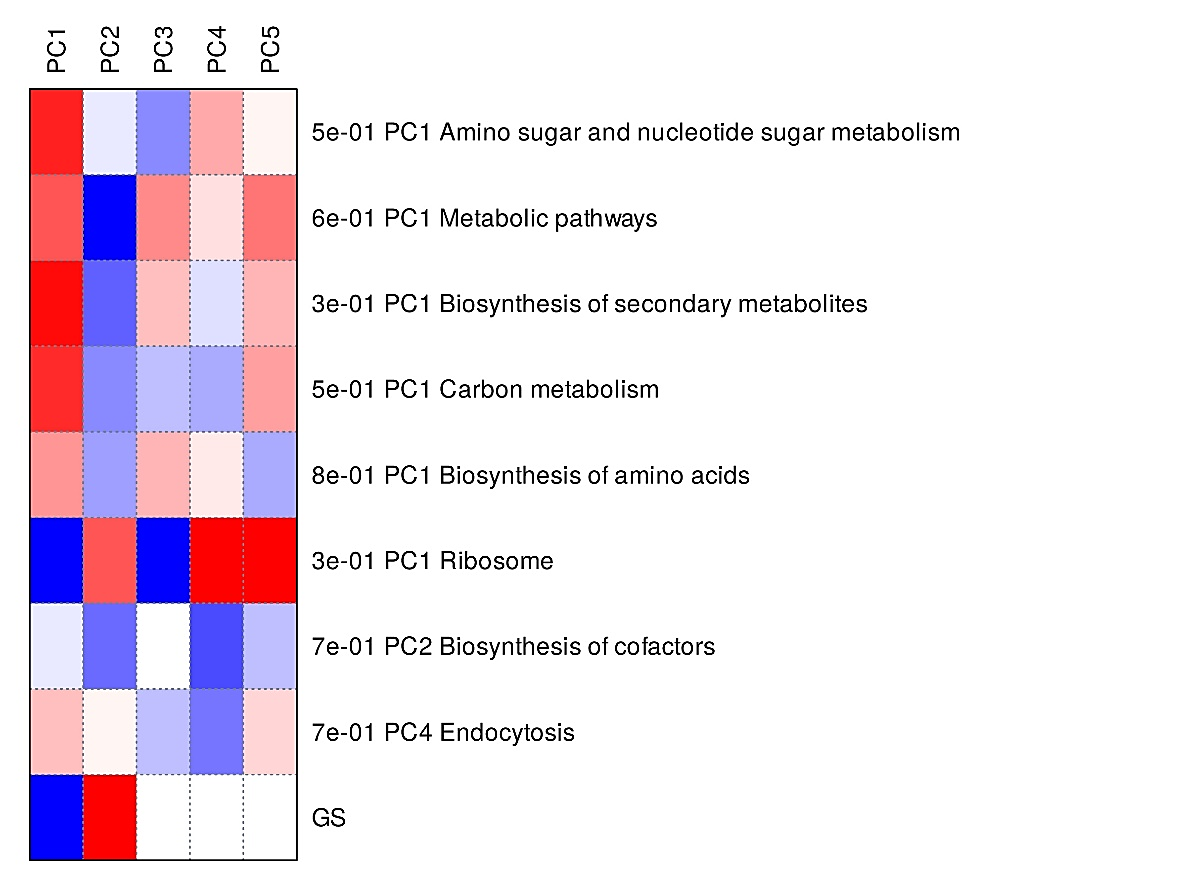


**Figure S6**


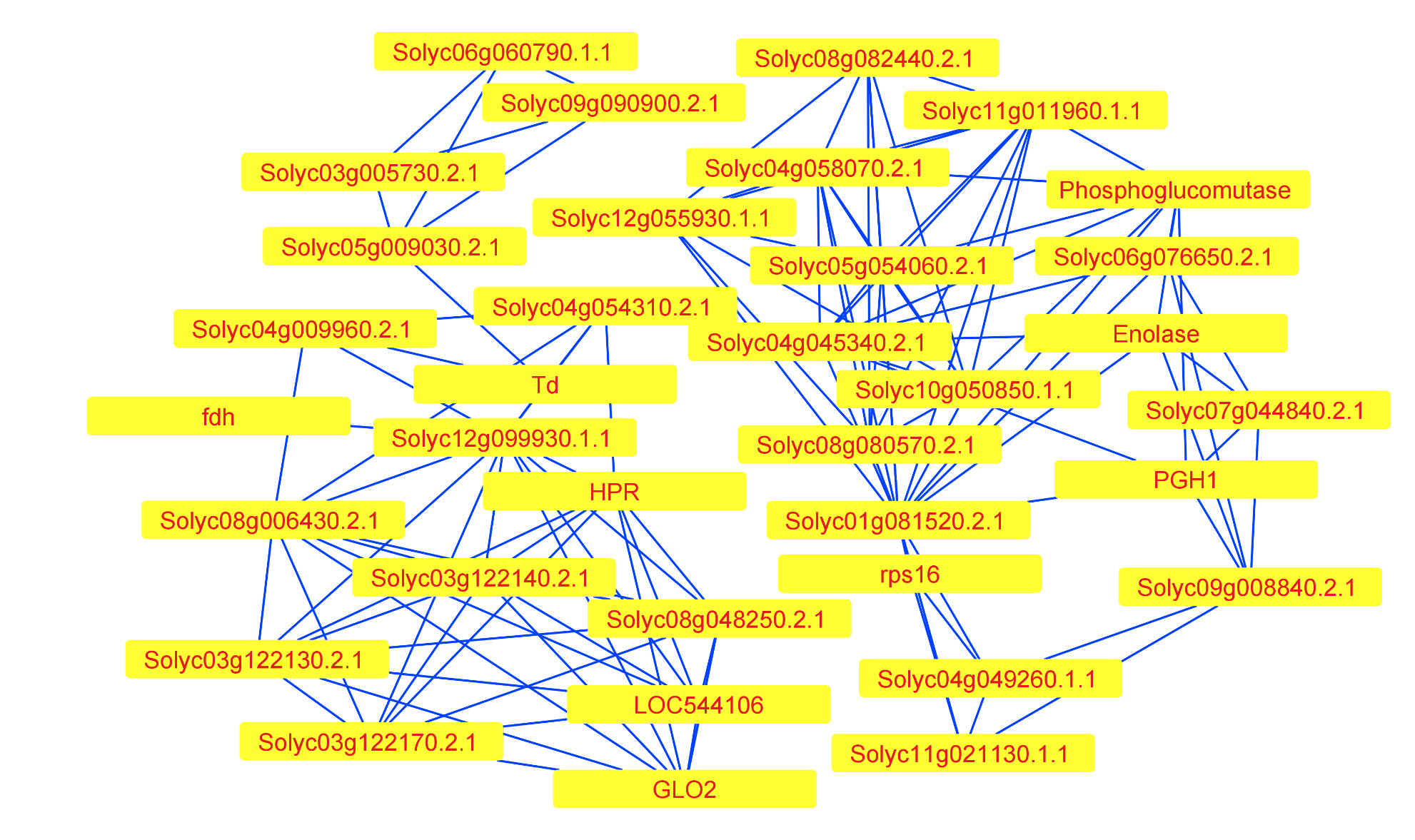


**Figure S7**

**
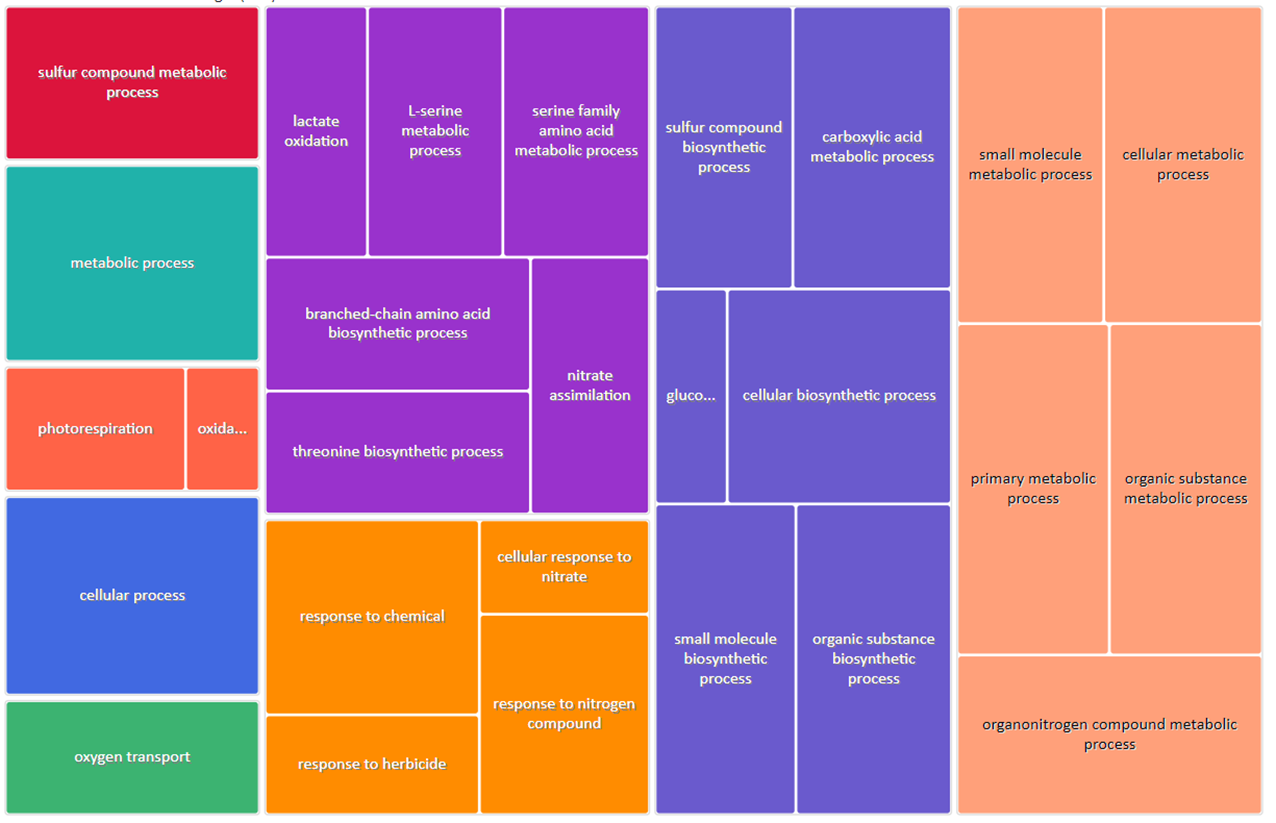
**

**Figure S8**

**
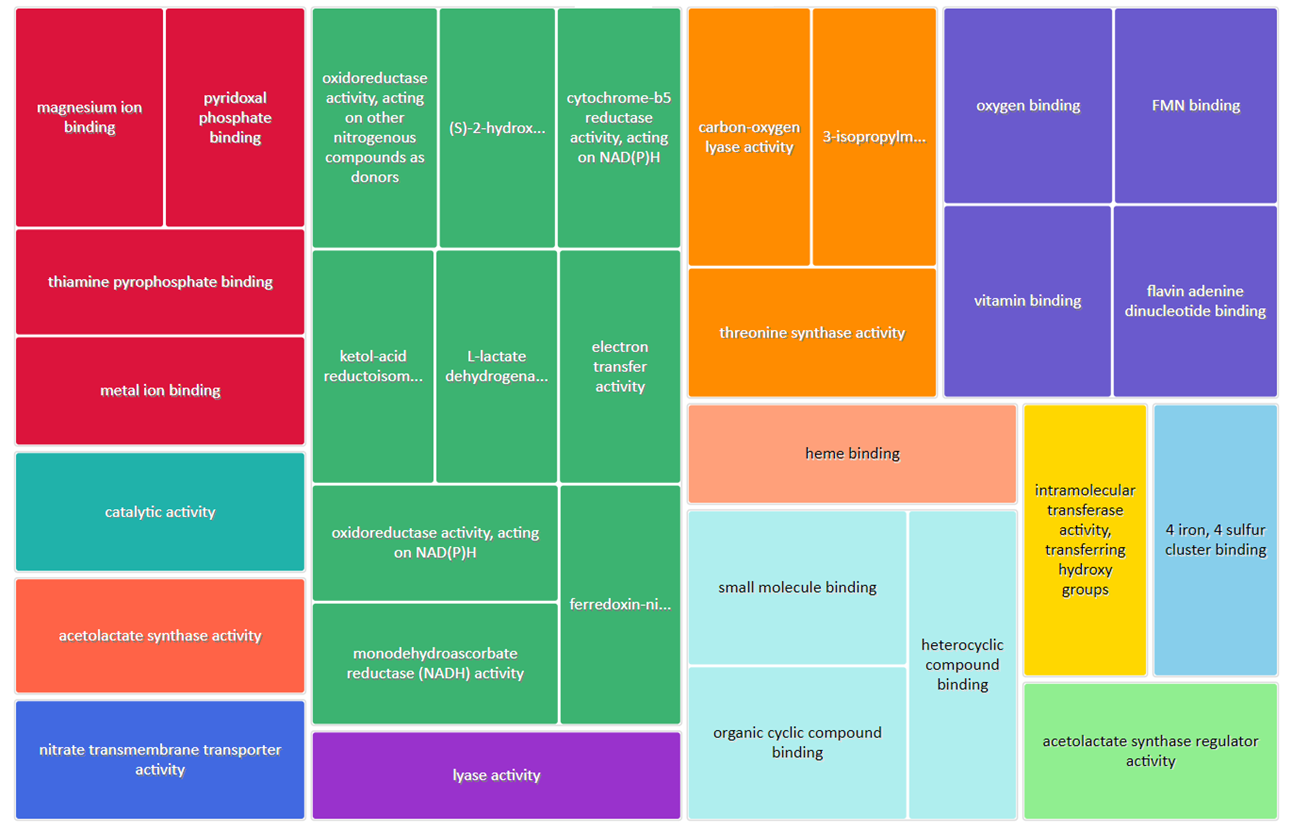
**

**Table S1**

|  | **PCA.1** | **PCA.2** | **PCA.3** | **PCA.4** | **PCA.5** | **MDS.x** | **MDS.y** | **tSNE.x** | **tSNE.y** |
| --- | --- | --- | --- | --- | --- | --- | --- | --- | --- |
| **C1** | -5.55748 | 2.361108 | -1.36865 | 0.743265 | -0.44514 | -0.00357 | 0.001439 | -92.1459 | -141.391 |
| **C2** | -4.83087 | 0.728724 | 1.74763 | 0.104422 | 0.926899 | -0.00269 | 0.00043 | -111.356 | -84.6016 |
| **C3** | -5.22136 | -3.15073 | -0.21776 | -0.85332 | -0.45577 | -0.00348 | -0.00187 | -142.36 | 42.00679 |
| **T1** | 5.032274 | 2.091745 | -1.2692 | -0.89008 | 0.479462 | 0.003086 | 0.001283 | 112.648 | -7.56452 |
| **T2** | 5.384056 | 1.110636 | 1.742054 | 0.036704 | -0.85868 | 0.003359 | 0.000631 | 116.1497 | 51.1188 |
| **T3** | 5.193379 | -3.14148 | -0.63408 | 0.859007 | 0.353232 | 0.003303 | -0.00191 | 117.0647 | 140.4316 |

**Table S2**

| **Biological Processes** |  |  |  |  |
| --- | --- | --- | --- | --- |
| **GO Term** | **Description** | **Count in Network** | **Strength** | **FDR** |
| GO:0019516 | Lactate oxidation | 6 of 8 | 2.68 | 2.27E-11 |
| GO:0009854 | Oxidative photosynthetic carbon pathway | 7 of 10 | 2.65 | 2.70E-13 |
| GO:0050665 | Hydrogen peroxide biosynthetic process | 3 of 6 | 2.51 | 7.07E-05 |
| GO:0032889 | Regulation of vacuole fusion, non-autophagic | 3 of 6 | 2.51 | 7.07E-05 |
| GO:0006011 | UDP-glucose metabolic process | 2 of 4 | 2.51 | 0.0038 |
| GO:0019761 | Glucosinolate biosynthetic process | 3 of 7 | 2.44 | 7.85E-05 |
| GO:0009098 | Leucine biosynthetic process | 4 of 17 | 2.18 | 1.10E-05 |
| GO:0009853 | Photorespiration | 9 of 39 | 2.17 | 4.85E-14 |
| GO:0019320 | Hexose catabolic process | 2 of 10 | 2.11 | 0.0135 |
| GO:0006012 | Galactose metabolic process | 4 of 24 | 2.03 | 3.32E-05 |
| GO:0009082 | Branched-chain amino acid biosynthetic process | 5 of 35 | 1.96 | 2.27E-06 |
| GO:0006006 | Glucose metabolic process | 4 of 48 | 1.73 | 0.00018 |
| GO:0006096 | Glycolytic process | 5 of 84 | 1.58 | 7.07E-05 |
| GO:0019318 | Hexose metabolic process | 7 of 130 | 1.54 | 1.01E-06 |
| GO:0046686 | Response to cadmium ion | 3 of 62 | 1.49 | 0.013 |
| GO:0009225 | Nucleotide-sugar metabolic process | 3 of 63 | 1.49 | 0.0134 |
| GO:0010951 | Negative regulation of endopeptidase activity | 3 of 67 | 1.46 | 0.0154 |
| GO:1901607 | Alpha-amino acid biosynthetic process | 6 of 198 | 1.29 | 0.00012 |
| GO:1901615 | Organic hydroxy compound metabolic process | 7 of 317 | 1.15 | 0.00011 |
| GO:0032787 | Monocarboxylic acid metabolic process | 13 of 610 | 1.14 | 5.72E-09 |
| GO:1901605 | Alpha-amino acid metabolic process | 7 of 352 | 1.11 | 0.00019 |
| GO:0019752 | Carboxylic acid metabolic process | 22 of 1189 | 1.07 | 5.52E-15 |
| GO:0016052 | Carbohydrate catabolic process | 6 of 350 | 1.04 | 0.0021 |
| GO:0006520 | Cellular amino acid metabolic process | 8 of 482 | 1.03 | 0.00013 |
| GO:0044281 | Small molecule metabolic process | 32 of 2011 | 1.01 | 4.23E-22 |
| GO:0055086 | Nucleobase-containing small molecule metabolic process | 8 of 501 | 1.01 | 0.00016 |
| GO:1901135 | Carbohydrate derivative metabolic process | 11 of 916 | 0.89 | 4.05E-05 |
| GO:0046394 | Carboxylic acid biosynthetic process | 7 of 606 | 0.87 | 0.0046 |
| GO:0005975 | Carbohydrate metabolic process | 13 of 1174 | 0.85 | 9.40E-06 |
| GO:0044283 | Small molecule biosynthetic process | 8 of 819 | 0.8 | 0.0042 |
| GO:0044419 | Interspecies interaction between organisms | 8 of 826 | 0.79 | 0.0044 |
| GO:0006952 | Defense response | 8 of 1209 | 0.63 | 0.0393 |
| GO:1901566 | Organonitrogen compound biosynthetic process | 10 of 1725 | 0.57 | 0.0228 |
| GO:0009058 | Biosynthetic process | 18 of 3641 | 0.5 | 0.00066 |
| GO:0044249 | Cellular biosynthetic process | 16 of 3343 | 0.49 | 0.0037 |
| GO:1901576 | Organic substance biosynthetic process | 15 of 3504 | 0.44 | 0.0178 |
| GO:0034641 | Cellular nitrogen compound metabolic process | 16 of 4335 | 0.37 | 0.0483 |
| GO:0071704 | Organic substance metabolic process | 39 of 11582 | 0.33 | 2.70E-06 |
| GO:0044237 | Cellular metabolic process | 36 of 10924 | 0.33 | 3.52E-05 |
| GO:0008152 | Metabolic process | 41 of 13465 | 0.29 | 1.15E-05 |
| **Molecular Function** |  |  |  |  |
| GO:0008891 | Glycolate oxidase activity | 3 of 3 | 2.81 | 1.01E-05 |
| GO:0003861 | 3-isopropylmalate dehydratase activity | 3 of 3 | 2.81 | 1.01E-05 |
| GO:0003983 | UTP:glucose-1-phosphate uridylyltransferase activity | 2 of 2 | 2.81 | 0.0011 |
| GO:0052854 | medium-chain-(S)-2-hydroxy-acid oxidase activity | 6 of 8 | 2.68 | 7.20E-11 |
| GO:0052853 | long-chain-(S)-2-hydroxy-long-chain-acid oxidase activity | 6 of 8 | 2.68 | 7.20E-11 |
| GO:0052852 | very-long-chain-(S)-2-hydroxy-acid oxidase activity | 6 of 8 | 2.68 | 7.20E-11 |
| GO:0051748 | UTP-monosaccharide-1-phosphate uridylyltransferase activity | 3 of 4 | 2.68 | 1.49E-05 |
| GO:0050486 | Intramolecular transferase activity, transferring hydroxy groups | 2 of 3 | 2.63 | 0.0018 |
| GO:0004459 | L-lactate dehydrogenase activity | 6 of 10 | 2.59 | 7.20E-11 |
| GO:0004634 | Phosphopyruvate hydratase activity | 3 of 5 | 2.59 | 2.26E-05 |
| GO:0004614 | Phosphoglucomutase activity | 2 of 4 | 2.51 | 0.0025 |
| GO:0003978 | UDP-glucose 4-epimerase activity | 3 of 7 | 2.44 | 4.60E-05 |
| GO:0008453 | Alanine-glyoxylate transaminase activity | 2 of 5 | 2.41 | 0.0034 |
| GO:0070569 | Uridylyltransferase activity | 4 of 13 | 2.3 | 2.49E-06 |
| GO:0016868 | Intramolecular transferase activity, phosphotransferases | 3 of 14 | 2.14 | 0.00023 |
| GO:0010181 | FMN binding | 6 of 42 | 1.96 | 3.53E-08 |
| GO:0016866 | Intramolecular transferase activity | 5 of 59 | 1.74 | 8.72E-06 |
| GO:0016836 | Hydro-lyase activity | 6 of 95 | 1.61 | 2.49E-06 |
| GO:0016616 | Oxidoreductase activity, acting on the CH-OH group of donors, NAD or NADP as acceptor | 9 of 231 | 1.4 | 3.53E-08 |
| GO:0016853 | Isomerase activity | 8 of 298 | 1.24 | 4.26E-06 |
| GO:0016491 | Oxidoreductase activity | 11 of 2216 | 0.5 | 0.0344 |
| **KEGG Pathway** |  |  |  |  |
| sly00660 | C5-Branched dibasic acid metabolism | 4 of 8 | 2.51 | 2.86E-08 |
| sly00290 | Valine, leucine and isoleucine biosynthesis | 5 of 25 | 2.11 | 1.39E-08 |
| sly00052 | Galactose metabolism | 10 of 56 | 2.06 | 2.26E-16 |
| sly00966 | Glucosinolate biosynthesis | 2 of 14 | 1.96 | 0.0024 |
| sly00630 | Glyoxylate and dicarboxylate metabolism | 9 of 75 | 1.89 | 2.38E-13 |
| sly00260 | Glycine, serine and threonine metabolism | 7 of 63 | 1.85 | 2.43E-10 |
| sly04146 | Peroxisome | 8 of 88 | 1.77 | 3.97E-11 |
| sly00520 | Amino sugar and nucleotide sugar metabolism | 10 of 132 | 1.69 | 4.34E-13 |
| sly01210 | 2-Oxocarboxylic acid metabolism | 4 of 61 | 1.62 | 3.39E-05 |
| sly01200 | Carbon metabolism | 15 of 271 | 1.55 | 1.09E-17 |
| sly00010 | Glycolysis / Gluconeogenesis | 7 of 132 | 1.53 | 2.61E-08 |
| sly01230 | Biosynthesis of amino acids | 11 of 225 | 1.5 | 1.60E-12 |
| sly00250 | Alanine, aspartate and glutamate metabolism | 2 of 51 | 1.4 | 0.0219 |
| sly00040 | Pentose and glucuronate interconversions | 4 of 113 | 1.36 | 0.0003 |
| sly00030 | Pentose phosphate pathway | 2 of 56 | 1.36 | 0.0249 |
| sly00500 | Starch and sucrose metabolism | 5 of 142 | 1.35 | 3.39E-05 |
| sly00230 | Purine metabolism | 3 of 96 | 1.3 | 0.004 |
| sly03018 | RNA degradation | 3 of 108 | 1.25 | 0.0052 |
| sly01110 | Biosynthesis of secondary metabolites | 27 of 1266 | 1.14 | 9.90E-23 |
| sly01100 | Metabolic pathways | 33 of 2330 | 0.96 | 4.16E-23 |
|  |  |  |  |  |

**Table S3**

| **Biological Processes** |  |  |  |  |
| --- | --- | --- | --- | --- |
| **GO Term** | **Description** | **Count in Network** | **Strength** | **FDR** |
| GO:0019419 | Sulfate reduction | 3 of 3 | 2.85 | 1.16E-05 |
| GO:0070814 | Hydrogen sulfide biosynthetic process | 5 of 6 | 2.77 | 1.20E-09 |
| GO:0000103 | Sulfate assimilation | 8 of 14 | 2.61 | 1.56E-14 |
| GO:0006108 | Malate metabolic proceess | 5 of 14 | 2.41 | 2.13E-08 |
| GO:0006097 | Glyoxylate cycle | 3 of 12 | 2.25 | 0.00022 |
| GO:2000904 | Regulation of starch metabolic process | 2 of 8 | 2.25 | 0.0107 |
| GO:0019344 | Cysteine biosynthetic process | 5 of 22 | 2.21 | 1.21E-07 |
| GO:0006099 | Tricarboxylic acid cycle | 9 of 50 | 2.11 | 1.74E-13 |
| GO:0000097 | Sulphur amino acid biosynthetic process | 6 of 47 | 1.96 | 4.81E-08 |
| GO:0010020 | Chloroplast fission | 2 of 19 | 1.87 | 0.0445 |
| GO:0042128 | Nitrate assimilation | 3 of 45 | 1.67 | 0.0062 |
| GO:0044272 | Sulfur compound biosynthetic process | 10 of 157 | 1.65 | 2.53E-11 |
| GO:0006790 | Sulfur compound metabolic process | 11 of 335 | 1.37 | 1.09E-09 |
| GO:0008652 | Cellular amino acid biosynthetic process | 7 of 223 | 1.35 | 8.30E-06 |
| GO:1901607 | Alpha-amino acid biosynthetic process | 6 of 198 | 1.33 | 8.30E-05 |
| GO:0009658 | Chloroplast organization | 4 of 154 | 1.26 | 0.0094 |
| GO:0043436 | Oxoacid metabolic process | 21 of 1294 | 1.06 | 4.87E-14 |
| GO:0019752 | Carboxylic acid metabolic process | 18 of 1189 | 1.03 | 1.66E-11 |
| GO:0046394 | Carboxylic acid biosynthetic process | 8 of 606 | 0.97 | 0.00036 |
| GO:0044262 | Cellular carbohydrate metabolic process | 6 of 450 | 0.97 | 0.0062 |
| GO:0044281 | Small molecule metabolic process | 22 of 2011 | 0.89 | 7.06E-12 |
| GO:0005975 | Carbohydrate metabolic process | 13 of 1174 | 0.89 | 1.82E-06 |
| GO:0044283 | Small molecule biosynthetic process | 9 of 819 | 0.89 | 0.00035 |
| GO:0032787 | Monocarboxylic acid metabolic process | 6 of 210 | 0.84 | 0.0251 |
| GO:0055114 | Oxidation-reduction process | 19 of 2372 | 0.75 | 7.09E-08 |
| GO:0044249 | Cellular biosynthetic process | 15 of 3343 | 0.5 | 0.0056 |
| GO:0044237 | Cellular metabolic process | 36 of 10924 | 0.37 | 6.21E-07 |
| GO:0071704 | Organic substance metabolic process | 33 of 11582 | 0.3 | 0.00 |
| GO:0008152 | Metabolic process | 37 of 13465 | 0.29 | 4.32E-05 |
| GO:0044238 | Primary metabolic process | 28 of 10505 | 0.28 | 0.0107 |
| GO:0009987 | Cellular process | 43 of 16999 | 0.25 | 3.50E-06 |
| **Cellular Component** |  |  |  |  |
| GO:0031351 | Integral component of plastid membrane | 2 of 11 | 2.11 | 0.0129 |
| GO:0010319 | Stromule | 2 of 14 | 2.0 | 0.0169 |
| GO:0009706 | Chloroplast inner membrane | 3 of 90 | 1.37 | 0.0187 |
| GO:0005759 | Mitochondrial matrix | 5 of 171 | 1.32 | 0.00083 |
| GO:0005777 | Peroxisome | 5 of 217 | 1.21 | 0.0018 |
| GO:0031969 | Chloroplast membrane | 4 of 245 | 1.06 | 0.0218 |
| GO:0009570 | Chloroplast stroma | 7 of 474 | 1.02 | 0.00083 |
| GO:0009941 | Chloroplast envelope | 5 of 403 | 0.94 | 0.0186 |
| **Molecular Function** |  |  |  |  |
| GO:0033741 | Adenylyl-sulfate reductase (glutathione) activity | 3 of 3 | 2.85 | 1.72E-05 |
| GO:0009973 | Adenylyl-sulfate reductase activity | 3 of 3 | 2.85 | 1.72E-05 |
| GO:0004781 | Sulfate adenylyltransferase (ATP) activity | 3 of 3 | 2.85 | 1.72E-05 |
| GO:0048307 | Ferredoxin-nitrite reductase activity | 2 of 2 | 2.85 | 0.0012 |
| GO:0047780 | Citrate dehydratase activity | 2 of 2 | 2.85 | 0.0012 |
| GO:0043546 | Molybdopterin cofactor binding | 2 of 2 | 2.85 | 0.0012 |
| GO:0008746 | NAD(P)+ transhydrogenase activity | 2 of 2 | 2.85 | 0.0012 |
| GO:0003994 | Aconitate hydratase activity | 2 of 2 | 2.85 | 0.0012 |
| GO:0030060 | L-malate dehydrogenase activity | 5 of  7 | 2.7 | 8.14E-09 |
| GO:0004020 | Adenylylsulfate kinase activity | 2 of 3 | 2.67 | 0.0015 |
| GO:0016661 | Oxidoreductase activity, acting on other nitrogenous compounds as donors | 3 of 5 | 2.63 | 2.59E-05 |
| GO:0030151 | Molybdenum ion binding | 2 of 10 | 2.15 | 0. 0.0079 |
| GO:0051539 | 4 Fe-4S cluster binding | 8 of 149 | 1.58 | 6.42E-08 |
| GO:0016667 | Oxidoreductase activity, acting on a sulfur group of donors | 5 of 148 | 1.38 | 0.00039 |
| GO:0020037 | Heme binding | 7 of 434 | 1.06 | 0.00039 |
| GO:0016491 | Oxidoreductase activity | 15 of 2216 | 0.68 | 3.99E-05 |
| GO:0046872 | Metal ion binding | 19 of 4527 | 0.47 | 0.00075 |
| GO:0003824 | Catalytic activity | 37 of 11224 | 0.37 | 4.38E-07 |
| GO:0043167 | Ion binding | 26 of 8276 | 0.35 | 0.0012 |
| **KEGG Pathway** |  |  |  |  |
| sly00920 | Sulfur metabolism | 10 of 36 | 2.29 | 3.08E-18 |
| sly00020 | Citrate cycle (TCA cycle) | 10 of 55 | 2.11 | 8.86E-17 |
| sly00450 | Selenocompound metabolism | 3 of 17 | 2.1 | 2.83E-05 |
| sly00630 | Glyoxylate and dicarboxylate metabolism | 10 of 75 | 1.97 | 1.14E-15 |
| sly00910 | Nitrogen metabolism | 3 of 31 | 1.84 | 0.00014 |
| sly01210 | 2-Oxocarboxylic acid metabolism | 5 of 61 | 1.76 | 5.09E-07 |
| sly00710 | Carbon fixation in photosynthetic organisms | 6 of 75 | 1.75 | 3.30E-08 |
| sly00620 | Pyruvate metabolism | 6 of 91 | 1.67 | 8.63E-08 |
| sly00270 | Cysteine and methionine metabolism | 6 of 112 | 1.58 | 2.50E-07 |
| sly00230 | Purine metabolism | 5 of 96 | 1.57 | 3.95E-06 |
| sly01200 | Carbon metabolism | 11 of 271 | 1.46 | 4.95E-12 |
| sly01230 | Biosynthesis of amino acids | 6 of 225 | 1.28 | 9.22E-06 |
| sly01100 | Metabolic pathways | 29 of 2330 | 0.94 | 1.14E-19 |
| sly01110 | Biosynthesis of secondary metabolites | 15 of 1266 | 0.92 | 3.18E-09 |
